# Chromosome-level genome assembly of *Amomum tsao-ko* provides insights into the biosynthesis of flavor compounds

**DOI:** 10.1093/hr/uhac211

**Published:** 2022-09-19

**Authors:** Ping Li, Genxiang Bai, Jiangbin He, Bo Liu, Junru Long, Taylan Morcol, Weiyao Peng, Fan Quan, Xinbo Luan, Zhenzhen Wang, Yi Zhao, Yunsheng Cha, Yuanyuan Liu, Juncai He, Lianzhang Wu, Yi Yang, Edward J Kennelly, Quan Yang, Lirong Sun, Zepeng Chen, Wanqiang Qian, Jian Hu, Jian Yan

**Affiliations:** Key Laboratory of Agro-Environment in the Tropics, Ministry of Agriculture and Rural Affairs, Guangdong Provincial Key Laboratory of Eco-Circular Agriculture, Guangdong Engineering Research Centre for Modern Eco-Agriculture, College of Natural Resources and Environment, South China Agricultural University, Guangzhou, 510642, China; Key Laboratory of Agro-Environment in the Tropics, Ministry of Agriculture and Rural Affairs, Guangdong Provincial Key Laboratory of Eco-Circular Agriculture, Guangdong Engineering Research Centre for Modern Eco-Agriculture, College of Natural Resources and Environment, South China Agricultural University, Guangzhou, 510642, China; Nujiang Green Spice Industry Research Institute, Lushui, Yunnan, 673100, China; Shenzhen Branch, Guangdong Laboratory of Lingnan Modern Agriculture, Genome Analysis Laboratory of the Ministry of Agriculture and Rural Affairs, Agricultural Genomics Institute at Shenzhen, Chinese Academy of Agricultural Sciences, Shenzhen, 518120, China; Key Laboratory of Agro-Environment in the Tropics, Ministry of Agriculture and Rural Affairs, Guangdong Provincial Key Laboratory of Eco-Circular Agriculture, Guangdong Engineering Research Centre for Modern Eco-Agriculture, College of Natural Resources and Environment, South China Agricultural University, Guangzhou, 510642, China; Department of Biological Sciences, Lehman College and The Graduate Center, City University of New York, Bronx, New York, 10468, USA; Key Laboratory of Agro-Environment in the Tropics, Ministry of Agriculture and Rural Affairs, Guangdong Provincial Key Laboratory of Eco-Circular Agriculture, Guangdong Engineering Research Centre for Modern Eco-Agriculture, College of Natural Resources and Environment, South China Agricultural University, Guangzhou, 510642, China; Key Laboratory of Agro-Environment in the Tropics, Ministry of Agriculture and Rural Affairs, Guangdong Provincial Key Laboratory of Eco-Circular Agriculture, Guangdong Engineering Research Centre for Modern Eco-Agriculture, College of Natural Resources and Environment, South China Agricultural University, Guangzhou, 510642, China; Key Laboratory of Agro-Environment in the Tropics, Ministry of Agriculture and Rural Affairs, Guangdong Provincial Key Laboratory of Eco-Circular Agriculture, Guangdong Engineering Research Centre for Modern Eco-Agriculture, College of Natural Resources and Environment, South China Agricultural University, Guangzhou, 510642, China; Key Laboratory of Agro-Environment in the Tropics, Ministry of Agriculture and Rural Affairs, Guangdong Provincial Key Laboratory of Eco-Circular Agriculture, Guangdong Engineering Research Centre for Modern Eco-Agriculture, College of Natural Resources and Environment, South China Agricultural University, Guangzhou, 510642, China; Department of Biological Sciences, Lehman College and The Graduate Center, City University of New York, Bronx, New York, 10468, USA; Nujiang Green Spice Industry Research Institute, Lushui, Yunnan, 673100, China; Key lab of Southwestern Crop Gene Resources and Germplasm Innovation, Ministry of Agriculture and Rural Affairs ,Yunnan Provincial Key Lab of Agricultural Biotechnology, Biotechnology and Germplasm Resources Institute, Yunnan Academy of Agricultural Sciences, Kunming, Yunnan, 650205, China; Nujiang Green Spice Industry Research Institute, Lushui, Yunnan, 673100, China; Nujiang Green Spice Industry Research Institute, Lushui, Yunnan, 673100, China; Nujiang Green Spice Industry Research Institute, Lushui, Yunnan, 673100, China; Department of Biological Sciences, Lehman College and The Graduate Center, City University of New York, Bronx, New York, 10468, USA; School of Chinese Materia Medica, Guangdong Pharmaceutical University, Guangzhou, 510006, China; State Key Laboratory of Organ Failure Research, Key Laboratory of Mental Health of the Ministry of Education, Guangdong-Hong Kong-Macao Greater Bay Area Center for Brain Science and Brain-Inspired Intelligence, Guangdong Province Key Laboratory of Psychiatric Disorders, Department of Neurobiology, School of Basic Medical Sciences, Southern Medical University, Guangzhou, 510515, China; Guangdong Provincial Tobacco Shaoguan Co. Ltd, Shaoguan, Guangdong, 512000, China; Shenzhen Branch, Guangdong Laboratory of Lingnan Modern Agriculture, Genome Analysis Laboratory of the Ministry of Agriculture and Rural Affairs, Agricultural Genomics Institute at Shenzhen, Chinese Academy of Agricultural Sciences, Shenzhen, 518120, China; Nujiang Green Spice Industry Research Institute, Lushui, Yunnan, 673100, China; Key lab of Southwestern Crop Gene Resources and Germplasm Innovation, Ministry of Agriculture and Rural Affairs ,Yunnan Provincial Key Lab of Agricultural Biotechnology, Biotechnology and Germplasm Resources Institute, Yunnan Academy of Agricultural Sciences, Kunming, Yunnan, 650205, China; Key Laboratory of Agro-Environment in the Tropics, Ministry of Agriculture and Rural Affairs, Guangdong Provincial Key Laboratory of Eco-Circular Agriculture, Guangdong Engineering Research Centre for Modern Eco-Agriculture, College of Natural Resources and Environment, South China Agricultural University, Guangzhou, 510642, China

## Abstract

*Amomum tsao-ko* is an economically important spice plant in the ginger family (Zingiberaceae). The dried ripe fruit has been widely used as spice and medicine in Southeast Asia due to its distinct flavor metabolites. However, there is little genomic information available to understand the biosynthesis of its characteristic flavor compounds. Here, we present a high-quality chromosome-level genome of *A. tsao-ko* with a total length of 2.08 Gb assembled into 24 chromosomes. Potential relationships between genetic variation and chemical constituents were analyzed by a genome-wide association study of 119 representative *A. tsao-ko* specimens in China. Metabolome and transcriptome correlation analysis of different plant organs and fruit developmental stages revealed the proposed biosynthesis of the characteristic bicyclononane aldehydes and aromatic metabolites in *A. tsao-ko* fruit. Transcription factors of 20 families may be involved in the regulatory network of terpenoids. This study provides genomic and chemical insights into the biosynthesis of characteristic aroma and flavor constituents, which can be used to improve the quality of *A. tsao-ko* as food and medicine.

## Introduction

**Figure 1:**
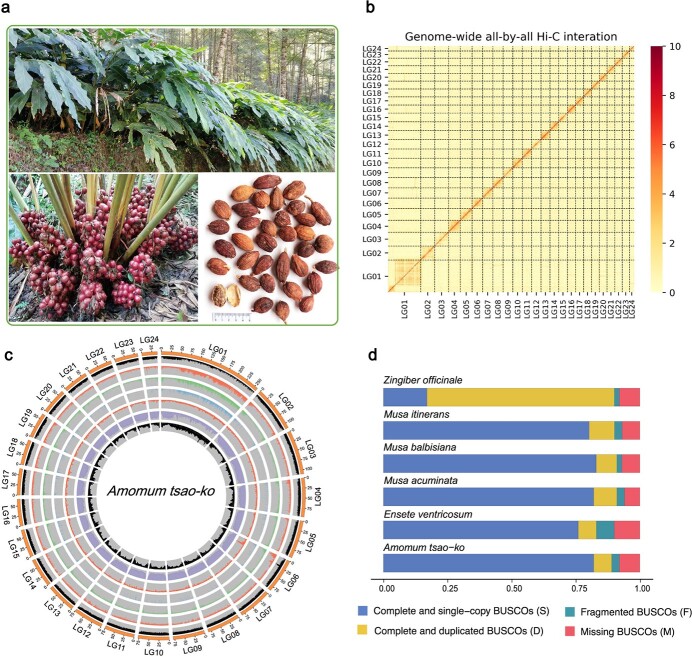
Overview of the *A. tsao-ko* genome assembly and features. **a***A. tsao-ko* plant, fresh fruits, and dried ripe fruits. **b** Hi-C map showing genome-wide all-by-all interactions between chromosomes. **c** Circular representation of characteristics of the 24 *A. tsao-ko* chromosomes (from inside to outside): SNP density, LTR retrotransposon density, LINE retrotransposon density, SINE retrotransposon density, DNA transposon density, gene density, GC content. **d** BUSCO analysis of *A. tsao-ko* and five other species. The BUSCO of *Zingiber officinale* genome showed large differences (73.3% duplicated genes) because of the diploid nature of the genome assembly [24].

*Amomum tsao-ko* Crevost et Lemarie (Fig. 1a), a perennial, evergreen, zingiberaceous plant, is mainly distributed in southwest China, northern Vietnam and other tropical and subtropical regions. As one of the most ancient natural spices, *A. tsao-ko* has been used in the indigenous diet of Asian countries for centuries [1, 2]. In China, the dried fruit of *A. tsao-ko* is commonly used as a traditional Chinese medicine for treating gastrointestinal fullness and pain, vomiting, and malaria [3]. The traditional Chinese medicine formula includes *A. tsao-ko* and has been used for the treatment of Covid-19 in China with good efficacy [4, 5]*. A. tsao-ko* is also a well-known culinary spice and condiment [6]. Due to its commercial and medicinal importance, *A. tsao-ko* is widely cultivated in Yunnan Province [7]. It is estimated that the annual Chinese production of the dried fruit of *A. tsao-ko* is nearly 1600 tons [8]. Over 90% of fruits were used culinarily and few were used medicinally or in other fields [9].

*A. tsao-ko* fruits contains essential oils, which give a characteristic aromatic and spicy odor and taste. The dominant essential oils include eucalyptol, geranial, geraniol, *trans*-2,3,3A,7A-tetrahydro-1H-indene-4-carbaldehyde (TI4C), (2E)-decenal, neral, and 4-indanecarbaldehyde [10–12]. The key aromatic compounds, such as eucalyptol, β-pinene, α-terpineol, and geranial, are found in other plants, and the biosynthetic pathway has been determined previously [13]. The pungent compound TI4C has been shown to evoke a refreshing and trigeminal sensation in the mouth [14]. It belongs to the hydrindane (bicyclo[4.3.0]nonane) class, only reported from *A. tsao-ko*^10^, and little is known about the biosynthesis of these bicyclononane aldehydes.

The fruits of *A. tsao-ko* have unique medicinal and flavor properties that are attributed to a variety of metabolites. *A. tsao-ko* possesses aromatic and spicy odor associated with its abundant terpenoids, which are synthesized from the universal five-carbon precursors isopentenyl diphosphate and dimethylallyl diphosphate [15, 16]. Terpene synthase (TPS) genes, responsible for the production of monoterpenes, have been isolated from many species [17]. The upstream synthases, including 1-deoxy-D-xylulose-5-phosphate synthase (DXS), 1-deoxy-D-xylulose-5-phosphate reductoisomerase (DXR), (E)-4-hydroxy-3-methylbut-2-enyl-diphosphate synthase (gcpE) and geranylgeranyl diphosphate (GPPS), contributing to geranyl pyrophosphate (GPP) biosynthesis have been identified [18]. Several TPS-related bioactive monoterpenoids in *Amomum* species have been characterized. The transcriptome sequencing of *Amomum villosum* at different ripening stages resulted in 10 TPS candidate genes, including *AvTPS1*, proven to catalyze the formation of α-pinene and β-pinene, and *AvTPS3*, shown to catalyze bornyl diphosphate from GPP [16]. TPS-gene functional analysis of *A. villosum* and *Amomum longiligulare* revealed that *AvBPPS* is the key gene for the biosynthesis of the bioactive monoterpenoids borneol and borneol acetate. Altering the expression of this gene may be useful for improving the medicinal quality and breeding of *Amomum* fruits [19].

Zingiberaceae is a family of flowering plants comprising roughly 1600 species of aromatic perennial herbs with horizontal or tuberous rhizomes and is divided into about 50 genera [20]. Many members are cultivated for use as spices, medicines, ornamentals, and cosmetics. Some species, like ginger (*Zingiber officinale*) and turmeric (*Curcuma longa*), are widely used in foods and traditional medicinal preparations [21, 22]. In recent years, the whole-genome sequencing analysis of Zingiberaceae plants has enhanced knowledge of genome evolution, gene regulation, and metabolite biosynthesis. For instance, the chromosome-scale reference genome for ginger revealed its evolution and the gingeroid biosynthetic pathway [23, 24]. Genome sequencing of turmeric provided insights into the evolution of the curcuminoid biosynthesis pathway [25]. *A. tsao-ko* is an economically important plant in the Zingiberaceae family; however, the lack of a high-quality reference genome for *Amomum* has limited the investigation of the biosynthetic pathway of flavor and bioactive metabolites.

Here we report a chromosome-scale genome assembly of *A. tsao-ko* and perform transcriptomic, metabolomic, and genome-wide association study (GWAS) analyses to reveal its characteristic metabolic regulatory network. Our goal is to understand the biosynthetic pathways of flavor chemical constituents (aromatic and pungent compounds) and trait biology to produce higher levels of these characteristic metabolites. The resulting metabolic network and genome dataset may be useful resources for the identification of key genes or regulators of important metabolites in Zingiberaceae and other spice plants. These findings may provide new opportunities for selective breeding to increase valuable *A. tsao-ko* metabolic traits.

## Results

### Complete genome assembly at the chromosome level in *A. tsao-ko*

In this study, a total of 202 Gb (100× coverage) Oxford Nanopore Technology (ONT) reads were generated from three libraries (Supplementary Data Table S1) in *A. tsao-ko*, which were used to assemble and polish the reference genome sequence by NECAT and Racon (https://github.com). The assembled genome included 1138 contigs with a total length of 2.08 Gb, a contig N50 of 4.8 Mb, and a contig N90 of 1.2 Mb (Table 1). The genome sizes are similar to the estimate for *A. tsao-ko* based on the distribution of *k*-mer frequencies (~2.0 Gb) and flow cytometry (1.98 ± 0.02 Gb) (Supplementary Data Fig. S1). Karyotype analysis of *A. tsao-ko* showed that the haploid *A. tsao-ko* genome consists of 24 chromosomes (Supplementary Data Fig. S2), consistent with previous karyotype research on Zingiberaceae [26]. Using Hi-C technology, a total of 1.98 Gb (95.2%) sequences were anchored and oriented into 24 linkage groups (Fig. 1b); the longest was 258.7 Mb and the shortest was 38.6 Mb (Fig. 1c, Supplementary Data Table S2). In the evaluation of completeness, 89.2% of Embryophyta core genes from OrthoDB (http://www.orthodb.org) were identified as complete in the reference gene set by Benchmarking Universal Single-Copy Ortholog (BUSCO) (Fig. 1d), comparable to the number for other published genomes (Supplementary Data Table S3).

The EVidenceModeler (EVM) pipeline was used to predict the protein-coding genes on the *A. tsao-ko* reference genomes. In total, 51 965 gene models were predicted in the assembled genome (Table 1). For functional annotation, a total of 92.9%, 68.9%, 92.0%, and 95.4% coding proteins were annotated by functional databases, including eggNOG, GO, Interpro, and UniProt (Supplementary Data Table S4). In the high-quality *A. tsao-ko* genome, >78.9% genome sequences consisted of transposable elements (Table 1), 62.5% of which are long terminal repeat retrotransposons (LTR-RTs), followed by unclassified transposable elements (13.8%). Notably, the most abundant LTR-RT family present in the genome was Copia, accounting for 71.1% of all LTR elements, followed by Gypsy (26.7%).

### Evolution of *A. tsao-ko*

**Figure 2:**
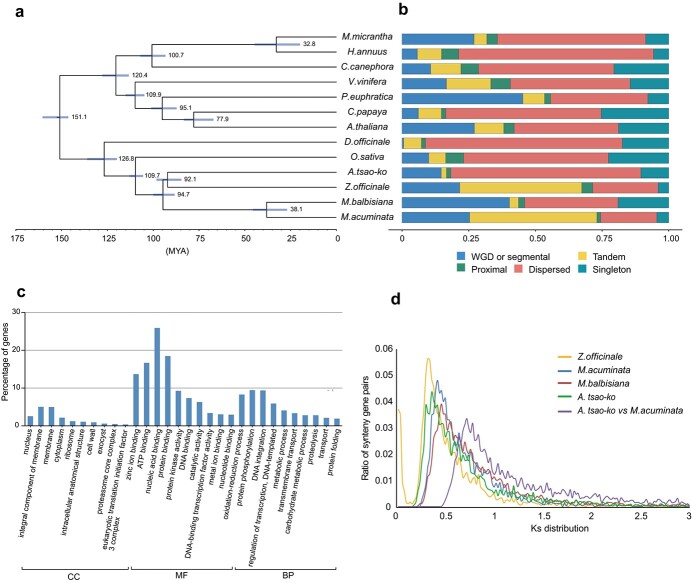
Evolutionary analysis of *A. tsao-ko*. **a** The phylogenetic tree with divergence times of 13 species. **b** Duplicated genes classified in 13 species. **c** GO annotation analysis of expansion ortholog group genes with results divided into three categories according to the GO domains (CC, cellular component; MF, molecular function; BP, biological process). **d***K*_s_ distribution of syntenic genes from *A. tsao-ko*, *Z. officinate*, *M. acuminata*, *Musa balbisiana*, and *A. tsao-ko* versus *M. acuminata*.

To gain insights into an evolutionary perspective of *A. tsao-ko*, a phylogenetic tree was constructed with PhyML based on 253 high-confidence single-copy ortholog groups of 13 species (*Arabidopsis thaliana*, *Carica papaya*, *Coffea canephora*, *Dactilon officinale*, *Helianthus annuus*, *Mikania micrantha*, *Musa acuminata*, *Musa balbisiana*, *Oryza sativa*, *Populus euphratica*, *Vitis vinifera, Z. officinale*, and *A. tsao-ko*) and estimated divergence time using MCMCTree (Fig. 2a, Supplementary Data Table S5). The results showed that *A. tsao-ko* diverged from the ancestors of *M. acuminate* and *M. balbisiana* ~78 million years ago (Mya) and from *D. officinale* ~131 Mya.

In the *A. tsao-ko* genome, there were 5448 singletons, 37 029 dispersed, 849 proximal, 1004 tandem, and 7635 WGD (whole-genome duplication) or segmental duplicated genes, which were much more than proximal duplicated genes in this genome (Fig. 2b). Utilizing pairwise protein sequence similarities, gene family clustering was conducted using orthoFinder. A total of 505 229 reference genes from the 13 species were clustered into 89 298 orthologous groups, among which 16 933 orthologous groups contained at least two genes each. A total of 20 415 orthologous groups were identified in the *A. tsao-ko* genome, in which 10 122 orthologous groups contained at least two genes each. There were 10 327 species-specific orthologous groups in *A. tsao-ko*, containing 10 766 genes. A total of 1189 ortholog groups showed significant expansion in the *A. tsao-ko* genome using the Z-test with *P*-value <.05, which contained 3813 genes. Annotating with gene ontology (GO), 53.2% (2028 of 3813) of the expanded genes were found to be mainly annotated in processes involved in nucleic acid binding, protein binding, ATP binding, and protein phosphorylation as well as in metabolic regulation (Fig. 2c). The distribution of synonymous substitutions per synonymous site (*K*_s_) in *A. tsao-ko* showed an obvious peak at ~0.36 (Fig. 2d), and peaks at similar *K*_s_ values were identified in other three Zingiberales species. But there was no obvious collinearity section in paralog analysis, suggesting that there was no recent WGD event.

**Table 1:** Summary of assembly and transposable elements of the *A. tsao-ko* genome.

**Genome assembly**	
Total length of contig (bp)	2 087 837 952
Maximum contig length (bp)	31 639 852
N50 length (bp)	4 783 640
N90 length (bp)	1 229 377
GC content (%)	40.89
Longest linkage groups (Mb)	258.7
Shortest linkage groups (Mb)	38.6
**Genome annotation**	
Transposable elements (%)	78.9
Gene models	51 965

### Potential relationship between metabolic profiling and TPS genes of *A. tsao-ko*

We collected 119 samples of *A. tsao-ko* from cultivated plots at different transects in Nujiang area (Supplementary Data Table S6). The ripe fruit of *A. tsao-ko* can be divided into four types (spherical, ellipse, cone, or spindle shaped) on the basis of the length-to-width ratio (Supplementary Data Fig. S3a). The spherical-shaped fruits contain more volatile constituents and higher aroma content (Supplementary Data Fig. S3b and c) than the other three fruit types. The volatile and non-volatile constituents and relative content (area under the curve) of the 119 samples were determined by GC–MS and LC–MS, and a total of 921 metabolites were detected (721 non-volatile and 200 volatile) (Supplementary Data Tables S7 and S8). Hierarchical cluster analysis of the GC–MS and LC–MS data revealed that the 119 samples from different geographic populations clustered into two major groups and seven subgroups (Supplementary Data Fig. S4), indicating differences in metabolites among the samples.

**Figure 3:**
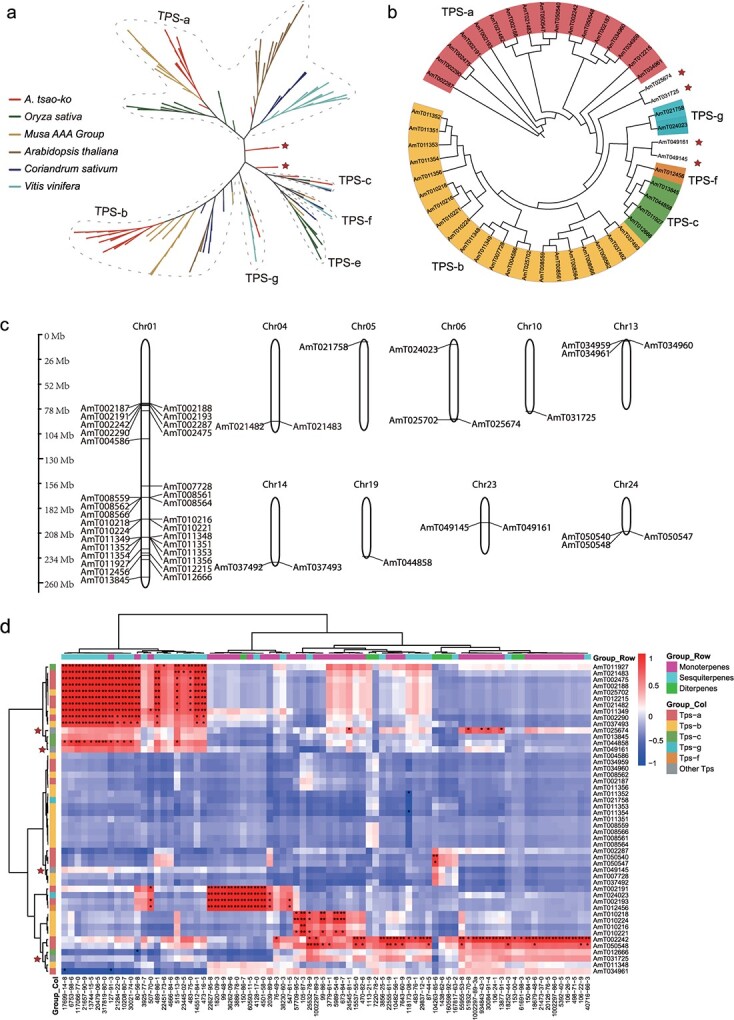
Metabolic profiling and TPS genes of *A. tsao-ko*. **a** Phylogenetic analysis of *A. tsao-ko* and five representative plant TPS gene families. The representative plants are shown as colored lines; star labels on the phylogenetic tree indicate the expanded genes. **b** TPS gene clusters detected in *A. tsao-ko*. **c** Chromosome distributions of TPS genes. **d** Correlation heat map of expression patterns of TPS genes and relative content of terpenoids in different organs and stages of ripeness. The compound name is presented by the CAS number from Supplementary Data Table S10.

Terpenoids are among the main volatile aromatic compounds, and originate from mevalonate (MVA) and mevalonate-independent (MEP) pathways. TPSs are responsible for the synthesis of various terpenoid compounds, which convert their precursors GPP, farnesyl diphosphate (FPP), and GGPP to monoterpenoids, sesquiterpenoids, and diterpenoids, respectively [16]. Based on the assembly genome, a total of 49 *AmTTPS*s were identified (Supplementary Data Table S9), and, using the phylogenetic tree topology, the *Arabidopsis* and coriander TPS genes were classified [17, 27]. The TPS genes of *A. tsao-ko* and five representative species can be divided into six subfamilies. The TPS-a and TPS-b subfamilies showed divergence between the species (Fig. 3a) and four *AmTTPS* (*AmT025674*, *AmT031725*, *AmT049161*, and *AmT049145*) were not attributed to any subfamilies (red stars in Fig. 3a and b), suggesting that the gene copies are produced by dispersed or segmental duplication after species divergence.

Phylogenetic analysis of 49 *AmTTPS* genes divided the *A. tsao-ko* samples into five subfamilies, with the TPS biosynthesis genes mainly distributed on chromosome 1 (Fig. 3c). Most of the *AmTTPS* genes were placed in the TPS-a (17 genes) and TPS-b (21 genes) subfamilies, primarily responsible for producing sesquiterpenoids and monoterpenoids, respectively. To reveal the TPS genes related to the synthesis of terpenoids, different organs and fruit ripening stages of *A. tsao-ko* were analyzed by RNA sequencing and GC–MS. Over 120 volatile compounds, including 80 terpenoids (Supplementary Data Table S10), were identified. The expression of 49 TPS genes varied in different fruit development stages; some TPS genes showed a relatively high expression level in October fruit (OF) and November fruit (NF) stages, while terpenoid accumulation increased in fruit ripening stages (Supplementary Data Fig. S5). The correlation heat map (Fig. 3d) revealed that the expression of 10 TPS genes (*AmT002191*, *AmT024023*, *AmT002193*, *AmT012456*, *AmT010218*, *AmT010224*, *AmT010216*, *AmT010221*, *AmT002242*, and *AmT050548*) correlated positively to monoterpenoids (*P* < .05 or .01). Eleven genes, *AmT011927*, *AmT021483*, *AmT002475*, *AmT002188*, *AmT025702*, *AmT012215*, *AmT021482*, *AmT011349*, *AmT002290*, *AmT037493*, and *AmT044858*, were significantly correlated to sesquiterpenoids. *AmT050540* were *AmT050547* were positively and significantly correlated to diterpenoids. The expression of TPS subfamilies was inconsistent with the content of terpenoid types in the significant areas (marked with asterisks) of the correlation heat map. For example, both TPS-a and TPS-b have been correlated with monoterpenoids and sesquiterpenoids. *AmT002242* (TPS-a) correlates highly with several monoterpenoids or sesquiterpenoids. Other TPS genes (four red star markers in Fig. 3b) correlated highly with monoterpenoids. *AmT025674* correlated significantly with three bicyclononane aldehydes, comprising 4-indanecarbaldehyde (51932-70-8), 5-indanecarbaldehyde (30084-91-4), and *cis*-2,3,3a,7a-tetrahydro-1H-indene-4-carbaldehyde (934843–43-3), as well as with two monoterpenoids, umbellulone (24545-81-1) and β-ocimene (13877-91-3). The correlation heat map of different organs and fruit ripening stages of TPS gene expression and terpenoid content provides important candidate genes that may regulate the biosynthesis of special flavor terpenoids in *A. tsao-ko*.

**Figure 4:**
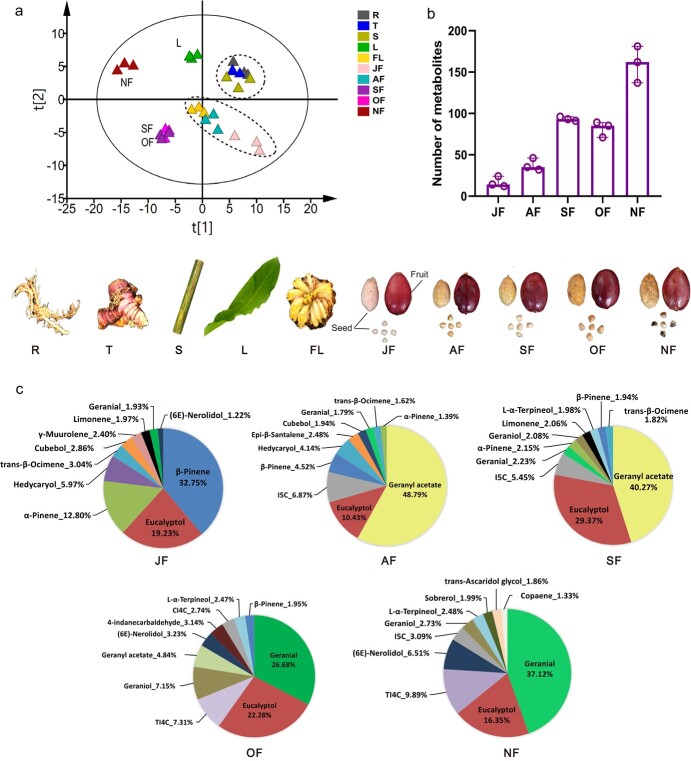
Content and distribution of volatile constituents in different organs and fruits of different ripening stages in *A. tsao-ko*. **a** PCA score plot of all the volatile constituents in different plant parts (*n* = 3). **b** Total number of aroma constituents in fruits collected at different stages of ripeness. **c** Percentage of top 10 volatile constituents at different stages of ripeness. (R, root; T, tuber; S, stem; L, leaf; FL, flower; JF, July fruit; AF, August fruit, …; NF, November fruit.).

### Correlation of characteristic flavor metabolites in different tissues and ripening stages with genetic variation of *A. tsao-ko* natural populations

*A. tsao-ko* compounds were divided into 13 classes, with alkanes, enols, aldehydes, and esters being the predominant metabolite classes. The percentage content of aromatic compound classes varied among different tissues; leaf and fruit displayed a wide variety of chemical constituents (Supplementary Data Fig. S6). Principal component analysis (PCA) showed that non-fruit parts and fruit ripening stages of metabolites were different (Fig. 4a). The root–tuber–stem formed one grouping. September fruit (SF) and OF had a similar score, indicating the similarity of their metabolites, while NF and leaf were separate from other samples, indicating that they contain different chemical constituents. The percentages of compound classes and numbers of individual metabolites changed significantly during fruit ripening (Fig. 4b). Aldehydes were the most abundant class in ripened fruits, increasing from 5.3% in July fruit (JF) to 58.7% in NF (Supplementary Data Fig. S6), which contributed to the increase in geranial and bicyclononane aldehydes. Other main aroma compounds, like eucalyptol, did not significantly change during ripening, while geranyl acetate actually decreased between August fruit (AF)/SF and OF/NF (Fig. 4c).

Most of the bicyclononane aldehydes have not been found in any other species and may be marker compounds characteristic of *A. tsao-ko* [11]. In this study, bicyclononane aldehydes, (Supplementary Data Fig. S7a), including TI4C, *cis*-2,3,3a,7a-tetrahydro-1H-indene-4-carbaldehyde (CI4C), 4-indanecarbaldehyde (4ICA), and 5-indanecarbaldehyde (5ICA), 2,3,3a,7a-tetrahydro-1H-indene-5-carbaldehyde (I5C), were detected and identified based on commercial standards and reference MS spectra. The bicyclononane aldehydes were only found in the fruit, and their content increased gradually as the fruit ripened, reaching highest levels of accumulation in ripe fruits collected in November (Supplementary Data Fig. S7b). These compounds were significantly higher in seeds relative to pericarp (Supplementary Data Fig. S7c). This indicates that bicyclononane aldehydes and pungent compounds are abundant in the mature seeds of *A. tsao-ko* fruit, which may reflect the evolutionary or breeding pressures experienced by this economically important species.

On the basis of chemical diversity profiling, 39 representative samples from the seven subgroups (Supplementary Data Fig. S4) were selected for re-sequencing and composition analysis. The average sequencing depth was 5.1×; the number of reads for Q20 was 94.6%, and the number of reads for Q30 was 56.5% (Supplementary Data Table S11). A total of 42 967 566 mutation sites were detected by BWA and SAMtools software [28, 29], accounting for 2% of the whole genome, of which 4 015 408 wereInDels. To further understand the genetic differences of the 39 representative samples, a phylogenetic tree was constructed using single-nucleotide polymorphisms (SNPs) from 39 *A. tsao-ko* samples and five Zingiberaceae species (*Alpinia zerumbet*, *Amomum villosum*, *Amomum maximum*, *Amomum yingjiangense*, *Amomum koenigii*). *A. tsao-ko* samples were placed in a group cluster and were different from the other five species, while *A. koenigii* was close to *A. tsao-ko* (Supplementary Data Fig. S8). A heat map of the contents of eight volatile compound is displayed in the outer phylogenetic tree with a bar plot in the middle displaying the total content of volatiles (Supplementary Data Fig. S8). Relative to the other Zingiberaceous plant species tested, *A. tsao-ko* had higher content of the characteristic bicyclononane aldehydes, and the bicyclononane aldehyde content varied significantly within the 39 representative samples. The total *A. tsao-ko* volatile compound content also varied at the population level, thereby suggesting considerable genetic diversity in this species.

### Flavor compounds and proposed biosynthetic pathway in *A. tsao-ko* fruit

Previous studies successfully synthesized TI4C in one step from (2*E*,8*E*)-2,8-decadienedial [30, 31], but the bicyclononane biosynthetic pathway in plants is not clear. The synthetic precursor (2*E*,8*E*)-2,8-decadienedial has not been reported to be a natural product and was not detected by GC–MS or LC–MS in any tissues of *A. tsao-ko* plants. The special flavor of *A. tsao-ko* fruit is associated with its unique composition of aromatic and pungent compounds (Fig. 4b). Most of these compounds are monoterpenoids, and TPS is the key enzyme involved in their biosynthesis in *A. tsao-ko*.

**Figure 5:**
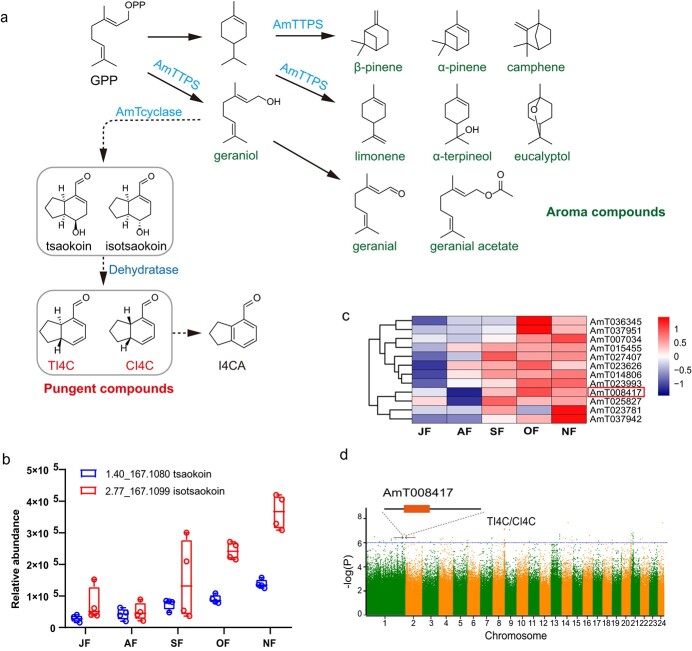
Metabolite variation in ripening stages of *A. tsao-ko* fruit and the proposed biosynthetic pathway of predominant flavor compounds. **a** Proposed biosynthetic pathway of main aroma and pungent compounds (bicyclononane aldehydes). GPP, geranyl diphosphate. AmTTPS, *A. tsao-ko* terpene synthases. AmTcylases, *A. tsao-ko* cyclases; TI4C, *trans*-2,3,3a,7a-tetrahydro-1H-indene-4-carbaldehyde; CI4C, cis-2,3,3a,7a-tetrahydro-1H-indene-4-carbaldehyde; 4ICA, 4-indanecarbaldehyde. **b** Relative content of tsaokoin and isotsaokoin at ripening stages of fruits. **c** Heat map of candidate genes relative to cyclases across different stages of fruit ripeness. **d** Manhattan plot of mGWAS results showing genetic associations for the pungent compound in *A. tsao-ko* fruit*.*

In an attempt to elucidate the biosynthesis of this pungent compound, we surveyed all structurally related compounds. Since all of the bicyclononane aldehydes in *A. tsao-ko*, including putative precursors, share a C_10_ skeleton, they likely all arise from the monoterpenoid pathway. A crude enzyme extract from ripened fruits was spiked and incubated with geraniol, which resulted in significantly higher levels of the pungent compound TI4C (Supplementary Data Fig. S9), suggesting that geraniol is a biosynthetic precursor to TI4C. The proposed biosynthetic pathway in *A. tsao-ko* begins with conversion of GPP to geraniol by *AmTTPS*, then to tsaokoin or isotsaokoin by a cyclase, and finally to the pungent compounds CI4C/TI4C by dehydratase (Fig. 5a). An unknown *A. tsao-ko cyclase* with a cyclase functional gene would be an essential enzyme to synthesize the bicyclo[4.3.0]nonane scaffold (Fig. 5a). The enolase, cyclase, and dehydratase genes in the *A. tsao-ko* genome are considered essential to the biosynthesis of this pungent compound. Four hidden Markov models (PF13243, PF13249, PF00175, PF08414) were used to identify the target sequences in the *A. tsao-ko* genome, in addition to Pearson correlation analysis of gene expression and the content of bicyclononanes in developmental stages of fruits to obtain 12 candidate genes. The functional annotation of the 12 candidate genes is listed in Supplementary Data Table S12. The relative expression of the 12 genes from transcriptome analysis and the content of tsaokoin and isotsaokoin had the same tendency, increasing gradually with fruit ripening, thus suggesting that those genes may regulate the formation of the bicyclononane scaffold of tsaokoin (Fig. 5b and c). The gene *AmT008417* is equally responsive in metabolite genome-wide association study (mGWAS) analysis to three bicyclononane aldehydes (TI4C, CI4C, and I5C) as phenotypic data (Fig. 5d, Supplementary Data Table S13). Thus, the gene *AmT00841*7, with functional annotation geranylgeranyl transferase type-2 subunit β, is a primary candidate for further testing of gene function.

### Contribution to formation of more terpenoids through transcription factors regulating the metabolite network

**Figure 6:**
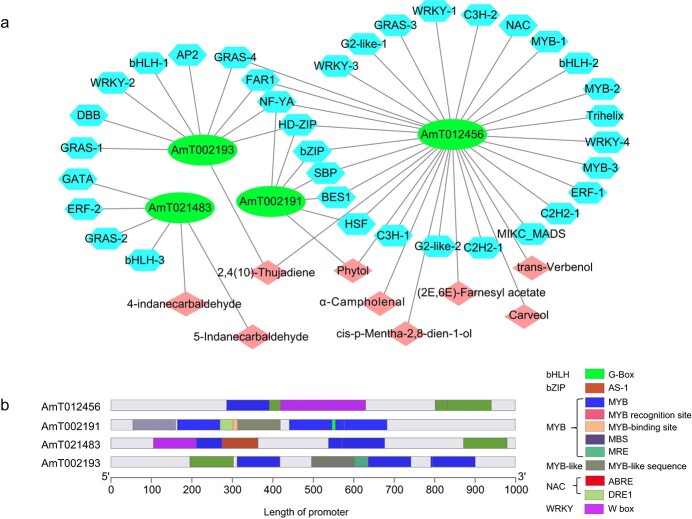
Transcriptional modulation of terpenoid biosynthetic genes. **a** Correlation network between structural genes (blue hexagons), transcription factors (green ellipses), and terpene compounds (brick-red diamonds) of terpenoid biosynthesis. The transcription factors were obtained by the correlation analysis (*r* > .8, *P* < .05). **b** Structure of the *cis*-regulatory element of gene promoters binding to transcription factors. Binding sites are color-coded by transcription factor.

Transcription factors regulate the transcription levels of secondary metabolite synthesis pathway genes [32]. The major transcription factor families, like NAC, ERF, bHLH, MYB, MYC, WRKY, and b-ZIP, have been found and associated with responses to biotic and abiotic stress in plants [33]. To study the relationship between transcription factors using metabolite networks, 2089 transcription factors from 58 transcription factor families were obtained from an annotation file of the *A. tsao-ko* genome (Supplementary Data Table S14). The transcriptomes of fruit at different stages of ripeness and across different plant tissues were evaluated using correlation heat maps (Supplementary Data Fig. S10, Supplementary Data Table S15). The expression levels of transcription factors, structural genes associated with terpenoid biosynthesis, and the content of terpenoids were subjected to correlation analysis (Supplementary Data Table S16), which resulted in the correlation network. The expression patterns of four structural genes were linked to the expression patterns of 35 transcription factors and were highly correlated with eight terpenoids, which may play an important role in terpenoid biosynthesis in *A. tsao-ko* fruit (Fig. 6a). The transcription factors included four *WRKY*s, four GRASs, three MYBs, three bHLHs, two C2H2s, and two G2-like, which belong to 20 families. Analysis of the *cis*-regulatory elements of the promoter showed that parts of the genes had binding sites corresponding to transcription factors (Fig. 6b), MYB binding sites including the MYB site, MBS, and MRE, WRKY binding site W-box, NAC binding sites ABRES and DRE1, bZIP binding site AS-1, and bHLH binding site G-box. Thus, the synthesis of terpenes is proposed to be regulated via these transcription factors. Interestingly, it was found that the structural gene *AmT021483* was highly correlated with bHLH (*AmT047586*), ERF (*AmT044753*), GRAS (*AmT005428*), and GATA (*AmT012452*) transcription factors, which relate to the synthesis of bicyclononane aldehydes (4-indanecarbaldehyde, 5-indanecarbaldehyde). The *cis*-regulatory elements of gene promoters showed that *AmT021483* contained AS-1, MYB, MRE, and W-box. These transcription factors may be involved in the regulation of the biosynthesis of special flavor bicyclononane aldehydes in *A. tsao-ko*.

## Discussion

*A. tsao-ko* is an economically important crop growing in tropical and subtropical humid forests [34]. The species has naturally undergone cross-pollination and cross-fertilization that has resulted in its rich phenotypic diversity [35]. The phenotypic traits of fruits collected from different regions in Yunnan Province have rich genetic variation [36]. Our results also found a rich diversity of *A. tsao-ko* fruit phenotypes and metabolites (Supplementary Data Fig. S3 and S4). Previous reports on *A. tsao-ko* focused mainly on the phenotype and DNA fragment molecular markers [37–39]. With increasing awareness given to the identification and protection of plant resources, research on the genetic diversity of *A. tsao-ko* has also increased (Supplementary Data Fig. S8). The complete *A. tsao-ko* genome provides sufficient data to resolve phenotypic and genetic variation. In addition, genetic diversity and environmental factors, such as altitude, ecological zones, weather, and climate, can also cause secondary metabolite variation via effects on biosynthetic pathways [40]. In this study, we found that the total number of volatile metabolites and the relative content of some of these metabolites in 119 *A. tsao-ko* fruit samples were positively correlated with higher altitude (Supplementary Data Fig. S11a–c). The fruit aromatic constituents, including 1,8-cineole, *trans*-citral, and the pungent compound (TI4C), decreased at high altitudes (>2000 m) (Supplementary Data Fig. S11d). This indicated that altitude can affect the biosynthetic pathway of aromatic constituents. Sequencing the *A. tsao-ko* genome has advanced ourunderstanding of its characteristic biochemistry and provided arationale to explain how environment may improve aroma andflavor metabolites through long-term evolutionary pressures.

The expression variations of GPP upstream related synthasegenes in the MEP and MVA pathways are essential to regulate flavor terpenoid biosynthesis. The rate-limiting enzymes DXS andDXR usually have different expression patterns, which are tissue-specific and related to plant growth and development stages [41, 42]. Hydroxymethylglutaryl-CoA reductase (HMGR) is considered to be the first rate-limiting enzyme in the MVA pathway and one of the important regulatory nodes in sesquiterpenoid synthesis [43]. Overexpression of hydroxymethylglutaryl-CoA synthase (HMGS) significantly increases plant growth and seed yield, suggesting itcan be used as an effective target in terpenoid metabolic engineering [44]. We analyzed all of the genes involved in the MEP and MVA pathways in the *A. tsao-ko* genome by local BLAST and hidden Markov models. Most of the genes were highly expressed in mature fruits, especially in OF (Supplementary Data Fig. S12). In the MVA pathway, HMGS contains five genes, and they arerelatively highly expressed in mature fruits (OF and NF). Similar trends were observed with other enzymes, like acetyl-CoA C-acetyltransferase (ACAT), HMGR, mevalonate kinase (MVK), diphosphomevalonate dacarboxylase (MVD), and farnesyl diphosphate synthase (FPPS) (Supplementary Data Fig. S12), indicating the precursor of sesquiterpenes increases in the ripening fruit of *A. tsao-ko*, thereby resulting in a greater variety of terpenoid accumulation in mature fruit. The genes involved in the MEP and MVA pathway exhibit a high level of fruit-development-specific expression also has observed in spice plant *Litsea cubeba* [45]. The diversity of terpenoids in the aroma of a plant is mainly determined by the TPS gene family. We identified 49 TPS genes belonging to six TPS subfamilies; a large number of TPS-a and TPS-b genes produce a great diversity of monoterpenes and sesquiterpenes. However, some TPS genes are less expressed in OF and NF. This may be related to the multifunctional enzymatic properties of some TPSs, which catalyze the formation of multiple terpenes from one precursor. For instance, germacrene D synthase, produced from FPP, was characterized in *Z. officinale* to catalyze the formation of several sesquiterpenes [46]. *HcTPS8* of *Hedychium coronarium* not only catalyzes GPP to produce linalool, but also converts FPP into 13 sesquiterpenes [47]. *ZSS1* can catalyze the formation of α-humulene and β-caryophyllene in *Zingiber zerumbet* [48]. Altogether, the biosynthetic pathway of flavor metabolites in *A. tsao-ko* is complex, and the upstream rate-limiting enzymes and TPS enzymes involved play important roles in their regulation.

*A. tsao-ko* has attracted attention as a functional food and medicine. Previous studies mainly focused on selected metabolite categories, such as organic acids [49], key aroma constituents [50], phenolic compounds [51, 52], diarylheptanoids [53], and terpenes [54]. A comprehensive and wide-scale metabolomics analysis of *A. tsao-ko* and its developmental process is necessary for developing molecular standards for *A. tsao-ko* quality assurance. This study simultaneously measured aromatic and non-volatile metabolites from different tissues and fruit growth stages using GC–MS and LC–MS, which provided a more comprehensive analysis of *A. tsao-ko* metabolites*.* By using a combination of metabolomics and genomics, we identified candidate genes and transcription factors associated with flavor-related volatiles. The use of molecular biotechnology to track flavor-associated compounds is emerging as the most advanced strategy for flavor improvement. A biosynthetic pathway of the uniquely pungent compound (TI4C) was proposed. Since *A. tsao-ko* is perennial, and since the fruit is typically harvested after 3 years, understanding key candidate genes that produce aromatic and spicy odor can help to predict which seedlings will produce high-quality fruits for use as a spice.

### Conclusions

We first present a high-quality chromosome-level genome of *A. tsao-ko* with a total length of 2.08 Gb assembled into 24 chromosomes. A complete genome provided sufficient informative sites for resolving phenotypic and genetic variation. Our study displayed the characteristic flavor-related regulatory genes, and the change in aromatic and pungent compounds during fruit ripening may serve as a foundation for the flavor biosynthesis of *A. tsao-ko*. The genome, large-scale transcriptome, and metabolome of *A. tsao-ko* generated in our study are valuable new resources for investigations of biology and breeding. These insights provide a better understanding of the evolution and phytochemistry of *A. tsao-ko* and related species, which can lead to improved bioengineering applications for producing more flavor substances from plants in the ginger family and beyond.

## Materials and methods

### Sample collection

*A. tsao-ko* samples were collected in the Nujiang area, Yunnan province. Different geographic populations of ripe fruits were sampled between 2019 and 2020 (Supplementary Data Table S6). The plant tissues (root, tuber, stems, leaves and flowers) were collected in July. The different ripening stages of fruits were collected in one garden from July to November (once a month to collect fresh fruits). The samples were collected and frozen at −80°C. Voucher specimens were identified by Dr Haijun Yang (South China Agricultural University) and have been deposited in the Herbarium of South China Agricultural University, China.

### Genome assembly

The software clean_adapter and clean_lowqual (https://github.com/fanagislab/DBG_assembly/tree/master/clean_illumina) was used to filter the Illumina raw reads with error rate <10^−3^. We filtered out the Oxford Nanopore Technology (ONT) reads that were <5 kb in length in this study. The high-quality ONT reads were corrected and assembled by NECAT (https://github.com/xiaochuanle/NECAT) using the default parameters. After assembly, we used the raw ONT reads to polish the assembled contigs with Racon (https://github.com/lbcb-sci/racon). Next, Illumina short reads were aligned to the assembled contigs using BWA-MEM, and base errors were polished by Pilon-v2.10 [55] using the parameters -fix bases, -nonpf, -minqual 20. Then, the Embryophyta gene set (odb9) was used to assess the integrity of the genome by BUSCO v3.0.2 [56].

The Hi-C sequencing raw reads were filtered using the following parameters: each read contained fewer than five bases from the adaptor, with average of base quality <19, and with unknown bases (N). The high-quality reads were aligned to the polished contigs using bowtie2 v2.2.3 [57], and we filtered out the invalid reads with HiC-Pro v2.7.8 [58], including unmapped pairs, dangling pairs, self-circles, and dumped reads. Clustering, ordering, and orienting were performed by the agglomerative hierarchical clustering algorithm (HCA) with LACHESIS [59]. For each cluster, the ordered contigs were oriented by building a weighted, directed acyclic graph (WDAG). The orientation of each contig in each linkage group was identified based on the maximum likelihood path according to WDAG. Then, each linkage group was cut into bins of 500 kb, and a heat map was constructed based on the interaction signals that were revealed by valid mapped read pairs between bins.

### Genome annotation

A *de novo* repeat library for *A. tsao-ko* was constructed by RepeatModeler (v1.0.4; http://www.repeatmasker.org/RepeatModeler/). Transposable elements were identified by RepeatMasker (v4.0.6; http://www.repeatmasker.org/). Tandem repeats were predicted using Tandem Repeats Finder v4.07b [60].

We used multiple methods for gene prediction in the *A. tsao-ko* genome. *Ab initio* prediction was performed using Augustus with default parameters [61]. A database containing non-overlapping protein sequence from closely related species was aligned to the *A. tsao-ko* genome sequences by genBlastA [62] (parameters: -e 1e-2 -g T -f F -a 0.5 -d 100 000 -r 10 -c 0.5 -s 0). Then, we used the software Genewise [63] to perform homology prediction. The pair-end reads of the transcriptome were aligned to the *A. tsao-ko* reference genome using TopHat, and then gene prediction was performed using Cufflinks [64]. Next, EVidenceModeler v 1.1.1 [65] was used to combine *de novo* predictions, homology alignments, and transcriptome read mapping. Then, the candidate protein-coding sequences were mapped by transcriptome data and functionally annotated based on the databases of UniProt [66] and InterProScan v5.16–55.0 [67]. Gene models were retained only if they had at least one point of supporting evidence from the homologous protein, protein domain, and gene expression.

For the gene functional annotation, we used the protein sequences of *A. tsao-ko* to align to the NCBI NR, UniProt, and EggNOG databases by Blastp v2.3.0+ with an E-value of 10^−5^. The functional pathway and classification were determined using the Kyoto Encyclopedia of Genes and Genomes (KEGG) database. InterProScan [67] was used to identify preliminary GO terms and functional domains to the gene models.

### Evolutionary analysis

Orthology of 13 species (Supplementary Data Table S5) and *A. tsao-ko* was inferred by OrthoFinder with default parameters. Single-copy orthologous genes for each species were selected to construct the phylogenetic tree. The protein sequences of single-copy genes were independently aligned by MAFFT v7.407 [68] and then concatenated into one super-sequence. A phylogenetic tree was constructed by maximum likelihood using RAxML v8.2.12 [69] with the best-fit model (GTR + I + G4) estimated by IQ-TREE multicore v1.5.5 [70].

The Bayesian relaxed molecular clock approach was used to estimate the species divergence time using the program MCMCTree of the PAML v4.9 package [71]. The calibration time interval (148–173 Mya) of the root was adopted from TimeTree (http://www.timetree.org). The genome synteny relationships were determined by the software MCscanX [72] with the cutoff of >10 homologous gene pairs in each syntenic block. To classify the different types of duplicate genes, the duplicate_gene_classifier of the MCscanX package was used in this study. The syntenic gene pairs were used to calculate the synonymous mutation rate (*K*_s_) using KaKs_Calculator 2.0 with default parameters [73].

### Transcriptome data analysis

RNA was isolated using an extraction kit (BioMarker, Beijing, China). The cDNA was synthesized from 3 μg of extracted total RNA using the PrimeScript™ RT Reagent Kit with genomic DNA Eraser (TaKaRa, Dalian, China) according to the manufacturer’s protocol. We used Cutadapt v1.18 [2] to remove adaptors from the raw reads and filter out low-quality reads. Clean reads were mapped to the reference genome of *A. tsao-ko* by HISAT2 v2.2.1 [74] (www.ccb.jhu.edu/people/infphilo) with parameter —min-intronlen 20. Read counts were calculated with featureCounts v2.0.1 [75] using SAM results from HISAT2, and FPKM (fragments per kilobase of transcript per million mapped reads) values were then calculated for every gene in the samples. Finally, the differentially expressed genes (DEGs) were identified with the DESeq2 R package v1.30.0 [76] based on |log_2_ (fold-change)| ≥ 1 and adjusted *P* value (*P* ≤ .05). GO enrichment analysis of DEGs was performed by cluster Profiler version 3.18.0 [77].

### Metabolomics analysis

Freeze-dried samples were ground to a powder under liquid nitrogen. Then the powder was extracted with dichloromethane for GC–MS analysis and methanol for LC–MS analysis. For more details, see the supplementary information. Raw data from both GC–MS and UPLC-QTOF-MS were processed with MS-DIAL software (v4.46) [78]. Briefly, raw MS data files were converted to ABF format. Then MS-DIAL was used for peak peaking, alignment, integration, and retention time correction according to optimized parameters (summarized in Supplementary Data Table S17). The resulting output data table of metabolites (i.e. peak areas for each RT-*m*/*z* pair in each sample) was subjected to further statistical analysis. The main volatile constituents were identified by comparing mass spectra and retention indices with reference standards, published literature, and the NIST library database. MS/MS spectra were compared with spectra from reference standards and from open databases, including METLIN, MassBank, ReSpect, GNPS, and BMDMS-NP, to identify non-volatile constituents. Multivariate analysis was performed using MetaboAnalyst 5.0, R and SIMICA 14.0 software (Umetrics), which provided the heat map, PCA, and orthogonal partial least squares discriminant analysis (OPLS-DA). The correlation heat map of genes and metabolites was made using the OmicStudio tools at https://www.omicstudio.cn/tool.

### Transcription factor identification and phylogenetic analysis

iTAK software (http://itak.feilab.net/cgi-bin/itak/index.cgi) was used to identify and classify transcription factors of the *A. tsao-ko* genome. The phylogenetic tree of the *A. tsao-ko* TPS synthesis gene transcription factors was constructed using the maximum likelihood criteria in IQ-TREE v2.1.4-beta [70] with the parameter -bb 1000.

## Acknowledgements

The work was funded by the National Natural Science Foundation of China (31970370, 31800283, 31560500), the Science and Technology Project of Nujiang Prefecture, Yunnan Province, China (2019CF1004, 2020CY004), the National Key R&D Program of China (2021YFC2600405, 2021YFC2600101) and the Science and Technology Planning Project of Guangdong Province, China (2019B030301007).

## Author contributions

Experimental design: J.Y., J.H., W.Q., L.S., and Q.Y. Genomic analyses: B.L. and J.L. Metabolomic analyses: P.L., T.M., W.P., F.Q., X.L., Y.Z., J.H., and Z.W. Transcriptome analyses and functional verification: P.L., G.B., J.L., J.H., X.L., and Y.Z. Sample collection and execution of experiments: P.L., J.H., Y.C., Y.L., J.H., L.W., and Y.Y. Manuscript writing: J.Y., P.L., G.B., and B.L. Project coordination and manuscript editing: J.L., E.J.K., T.M., Z.C., Q.Y., L.S., W.Q., J.H., and J.Y.

## Data availability

All data supporting the findings are available in the paper and supplementary information files. Genome assemblies have been deposited in the China National GeneBank DataBase (CNGBdb, https://db.cngb.org/search/project/CNP0003772/) with CNGB Project ID CNP0003772.

## Conflict of interest

The authors declare no competing interests.

## Supplementary data

Supplementary data is available at *Horticulture Research* online.

## Supplementary Material

Web_Material_uhac211
