# Supplementary material for: Chromosome-level genome assembly of Amomum tsao-ko provides insights into the biosynthesis of flavor compounds
Source: Hortic Res. 2022 Sep 19;9:uhac211. doi: 10.1093/hr/uhac211 (PMC9719038; doi:10.1093/hr/uhac211)
Supplement: Web_Material_uhac211 [file web_material_uhac211.zip › Supplementary Figures S1-S12_R1.pdf]

a

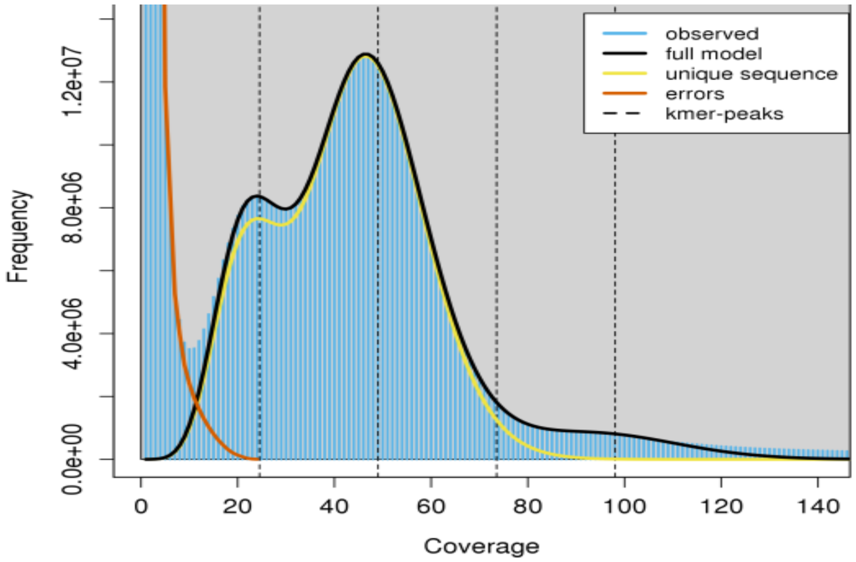

b

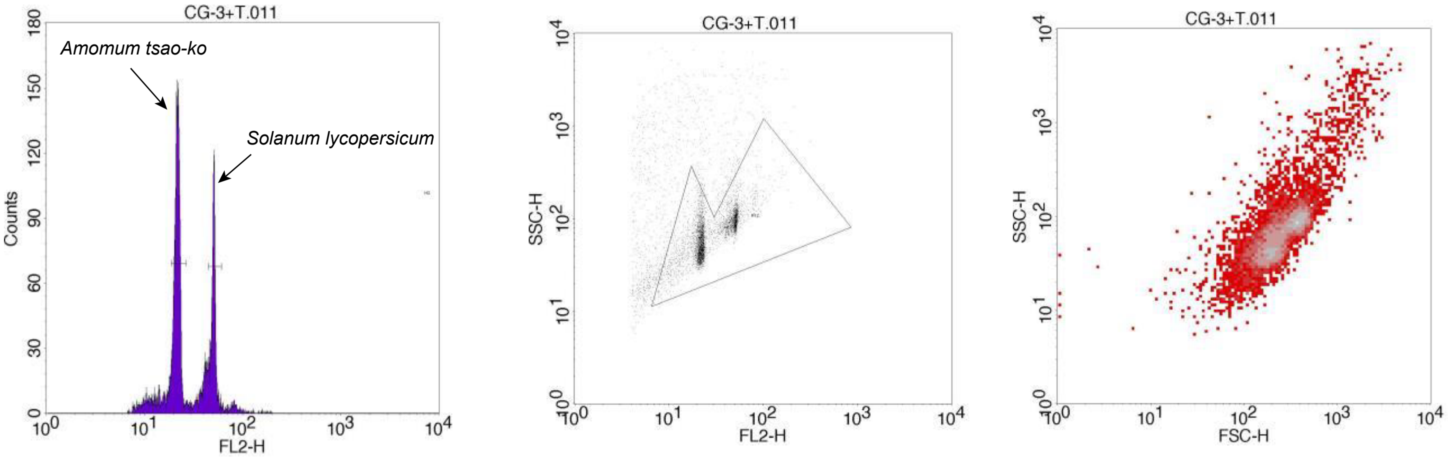

Supplementary Fig. 1 Assessment of genome size based on K-mers methods and flow cytometry. a, Distribution of 17-mer frequency in the sequence reads. The estimated genome size of A. tsao-ko was ~2.00 G. **b**, Flow cytometric analysis of *A. tsao-ko* leaf tissues.

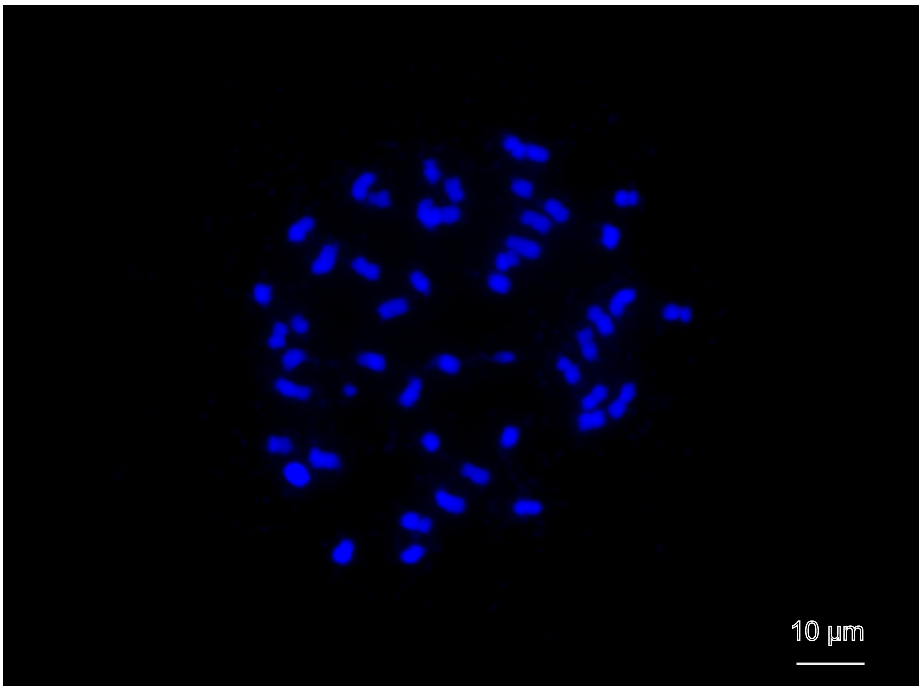

Supplementary Fig. 2 Fluorescence microscopy results of *A. tsao-ko* somatic chromosomes.

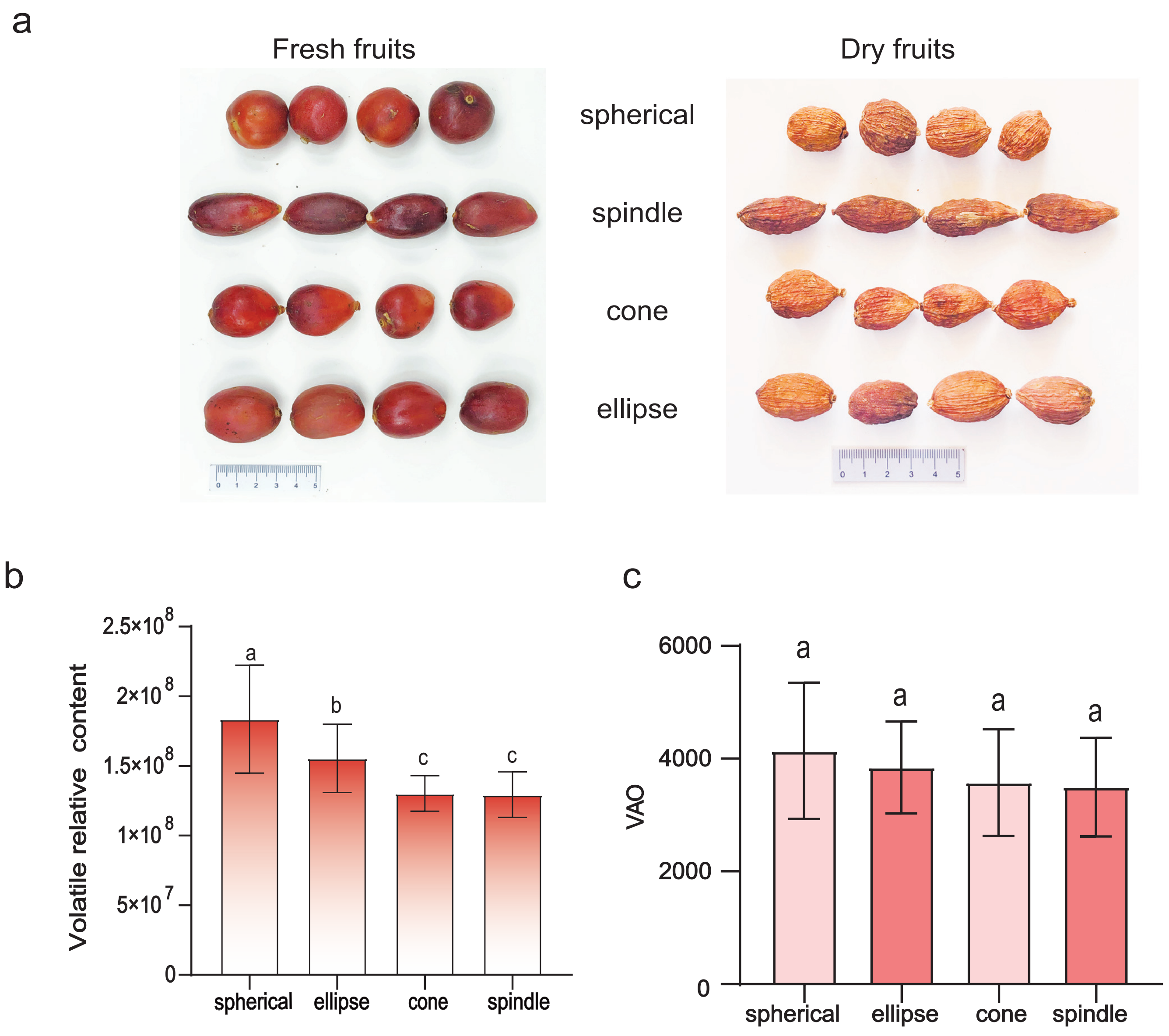

Supplementary Fig. 3 Fruit type and aroma value of *A. tsao-ko*. a, Fruit type can be divided into four types (spherical, ellipse, cone, spindle) . **b**, Relative total volatile content of four fruit types (area under curve). Mean S.D., of N=5, Dunnett's test relative to spherical group. **c**, Total aroma values from volatile constituents were calculated aroma threshold analysis. Odor Activity Value (OAV) was calculated according to OAV=C/OT, where C was the concentration of the compound and OT was its Ordor threshold. OAV represents contribution to ordor, higher OAV means more contribution to the flavor of sample.

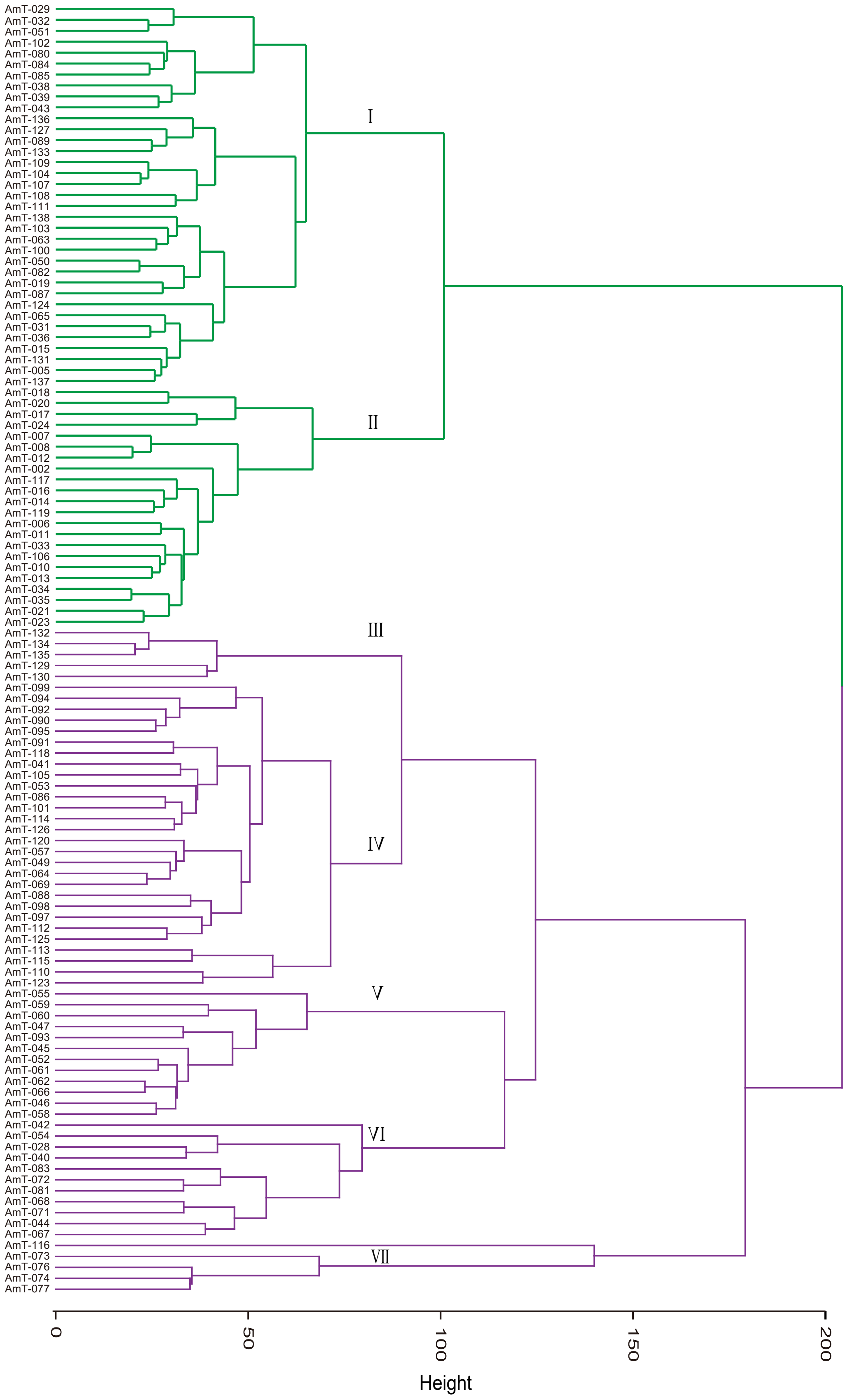

Supplementary Fig. 4 Hierarchical cluster analysis (HCA) of metabolites content of 119 A. tsao-ko fruits collected in Nujiang area. It was divided into two major groups and seven subgroups based on the linkage distance (height) of tree plot.

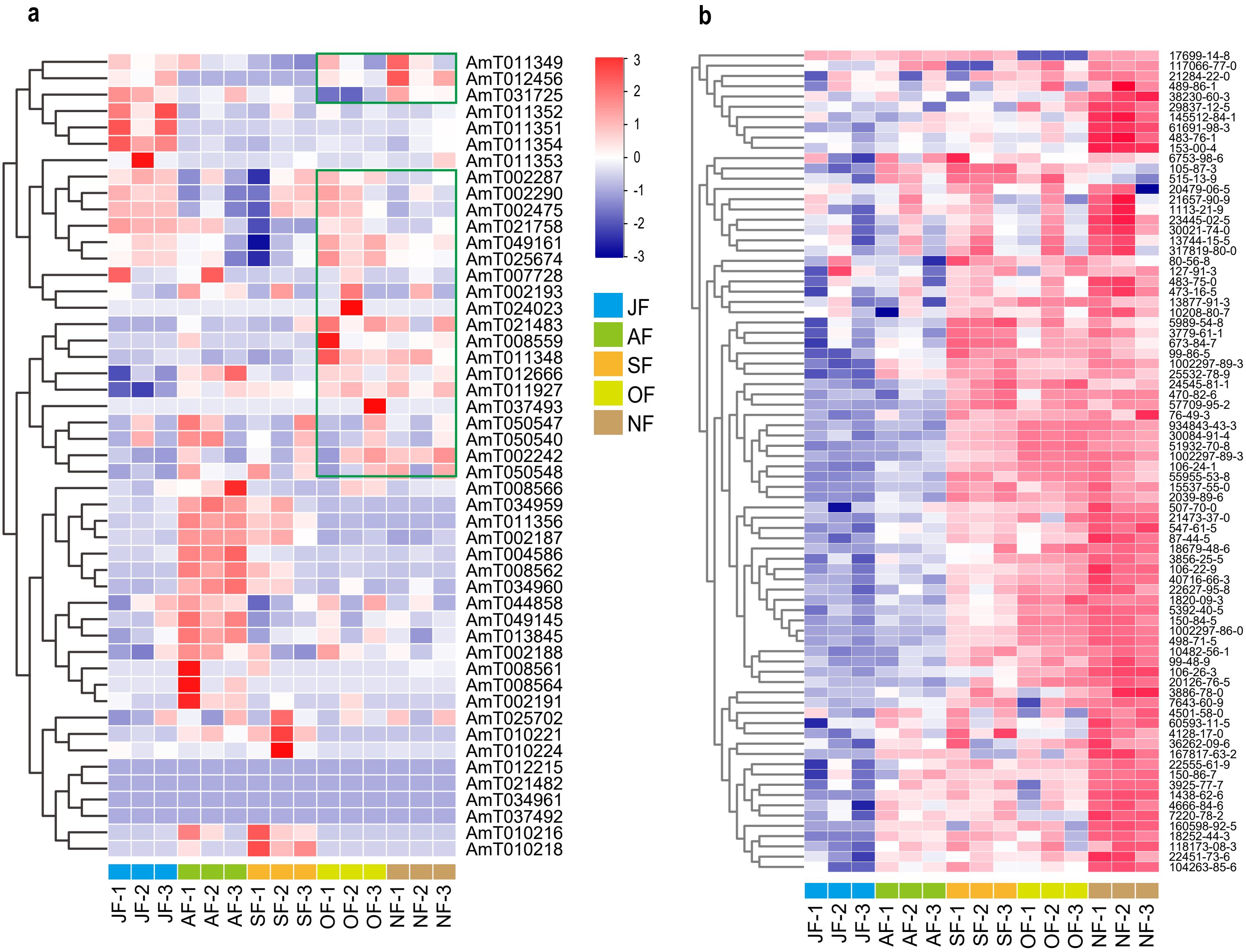

Supplementary Fig. 5 The expression pattern of TPS genes and the content of terpenes across different stages of ripeness. **a**. Heatmap of expression patterns of TPS genes. The green box marked are high expression of OF or NF. **b**. Heatmap of relative content of terpenoids. The compound name was presented by CAS No. from supplementary Table 10. (JF: July fruit, …, NF: November fruit, three replicates per sample group).

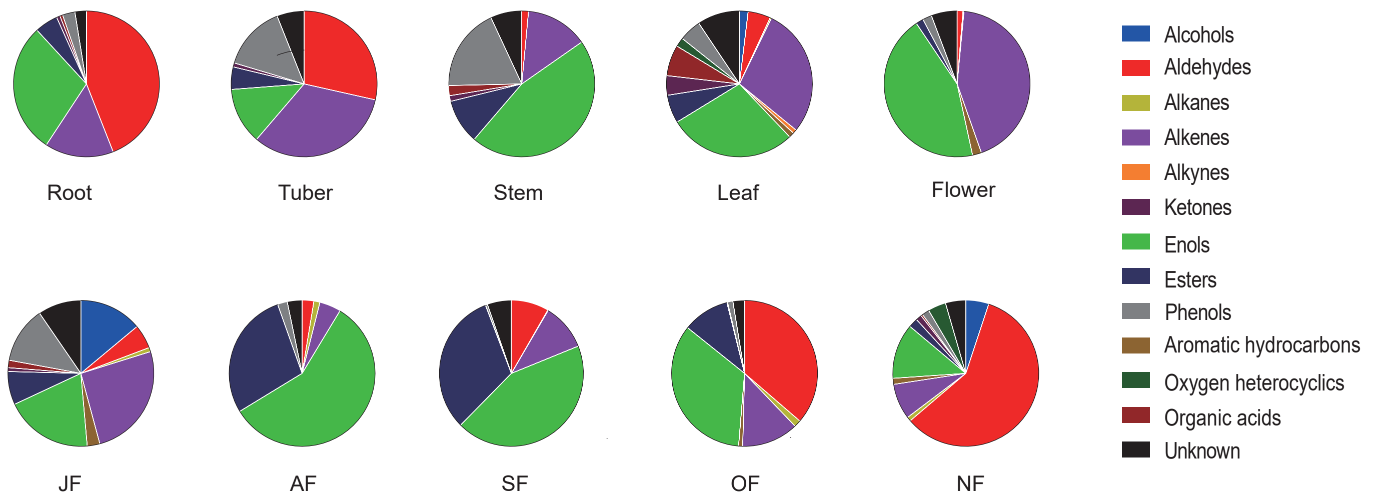

Supplementary Fig. 6 Relative proportion of various classes of volatile compounds in different plant parts and across different stages of ripeness (JF: July fruit, …, NF: November fruit), five replicates per sample group.

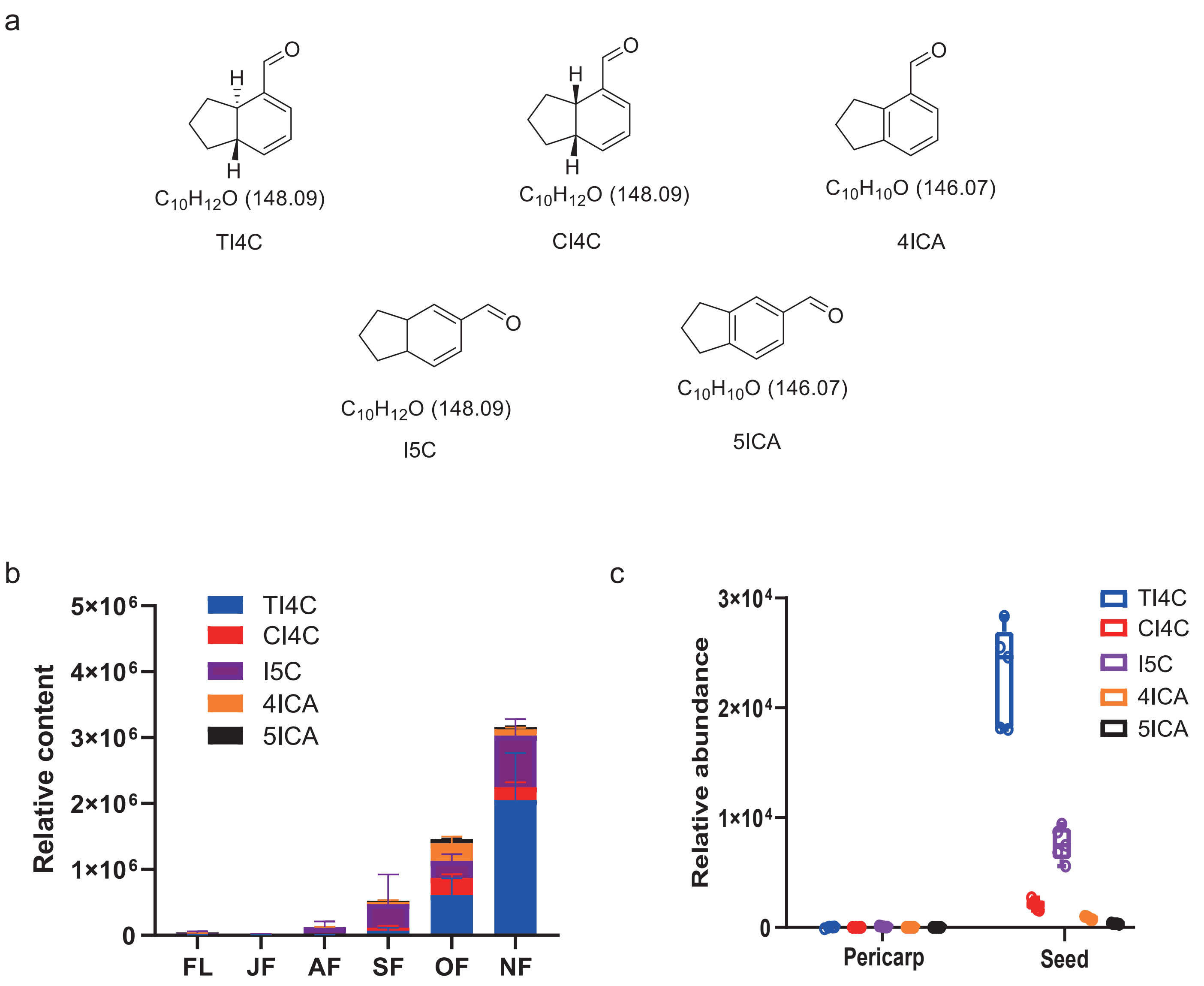

Supplementary Fig. 7 Bicyclononane aldehydes content in *A. tsao-ko* fruit at different stages of ripeness and within different tissues. a, Chemical structures of five bicyclononane aldehydes. **b**, Relative content of these five bicyclononane aldehydes in flowers and in fruit at different stages of ripeness. **c**, Comparative analysis of relative content of bicyclononane aldehydes between pericarp and seed. TI4C: trans-2,3,3a,7a-tetrahydro-1H-indene-4-carbaldehyde, CI4C:cis-2,3,3a,7a-tetrahydro-1H-indene-4-carbaldehyde; I5C: 2,3,3a,7a-tetrahydro-1H-indene-5-carbaldehyde; 4ICA: 4-indanecarbaldehyde, 5ICA: 5-indanecarbaldehyde. FL: flower,JF: July fruit, AF: August fruit,…, NF: November fruit.

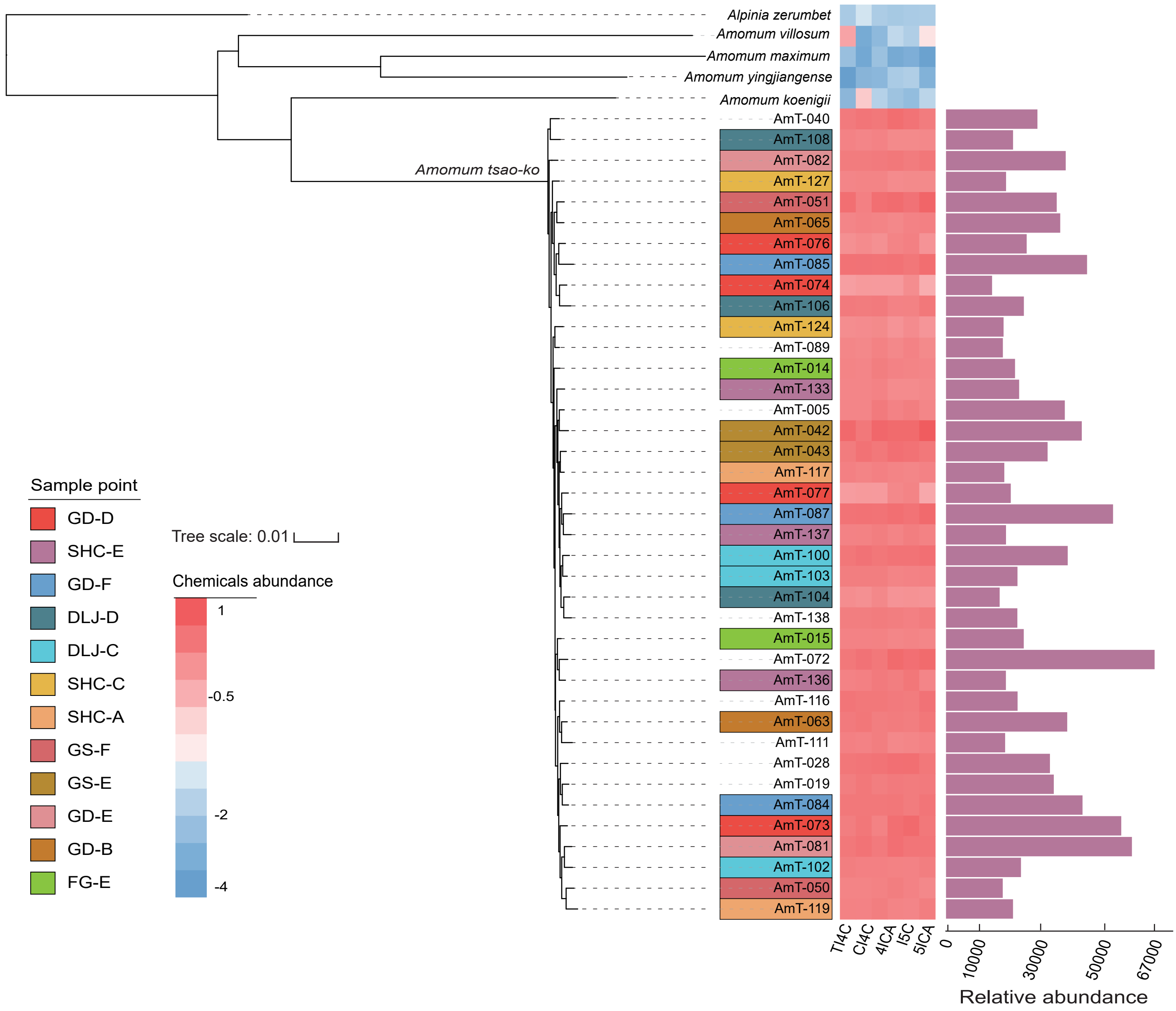

Supplementary Fig. 8 Phylogenetic tree of 39 *A. tsao-ko* samples and 5 other Zingiberaceous species. Two or more samples of located at the same sampling point are marked with same colors, no color indicate single sample point. The pink and blue heat map shows relative content of characteristic *A. tsao-ko* bicyclononane aldehydes, and the purple bar plot shows the total content of volatile aromatic in *A. tsao-ko*.

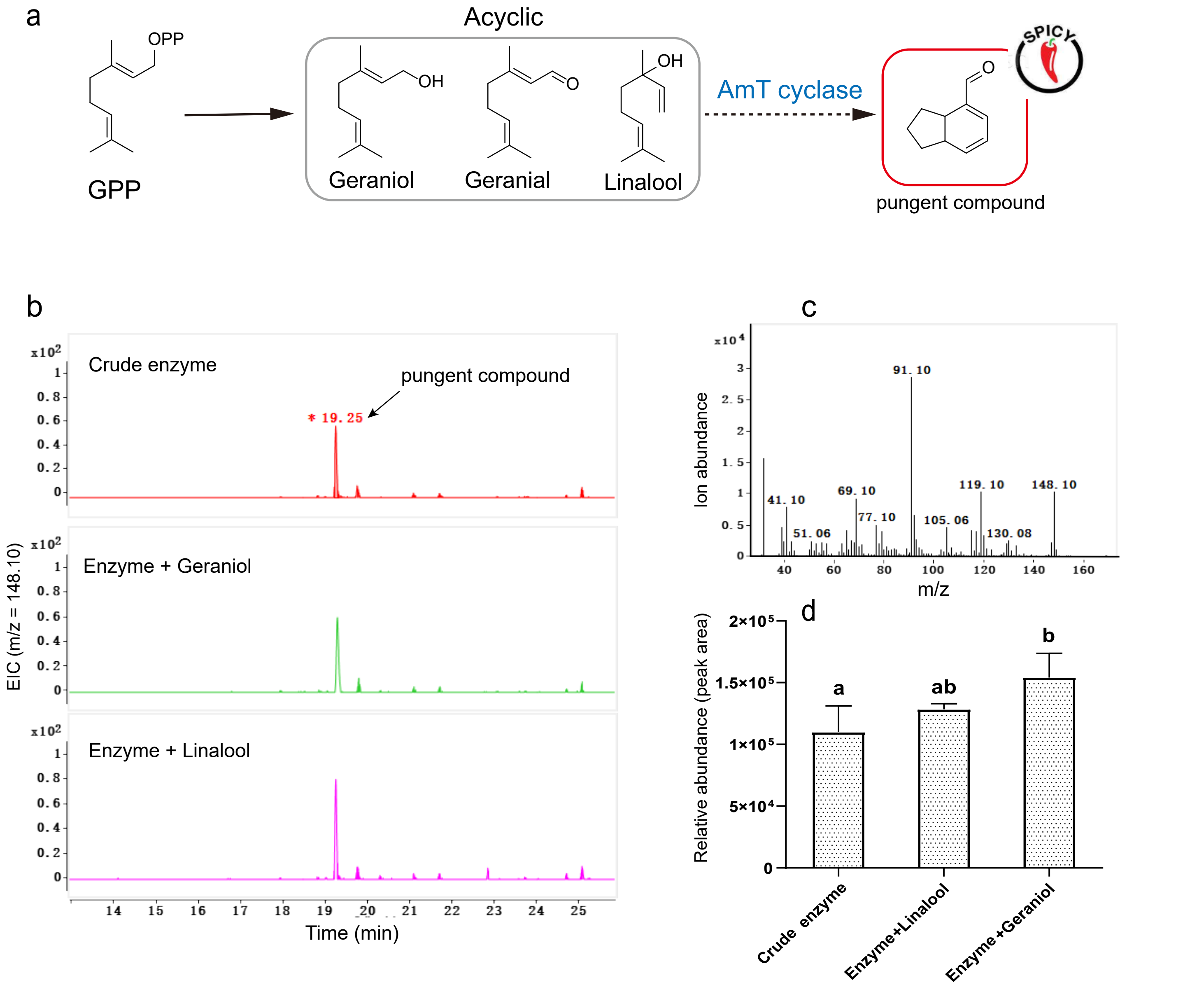

Supplementary Fig. 9 GC-MS demonstrate the content of pungent compound correlate with geraniol in vitro enzymatic activity. a, The hypothesized biosynthesis pathway of pungent compound. **b**, The extract ions chromatograph (EIC) at 148.1 m/z from crude enzyme of fresh *A. tsao-ko* fruit, crude enyzme added geraniol and linalool in vitro assays. Peak at 19.25 min was pungent compound (TI4C/CI4C). **c**, Mass spectrum of target peak at 19.25 min. **d**, The relative abundance of peak area at 19.25 min. Statistical significance (n = 3) determined using one-way ANOVA with Duncan's multiple comparison test. Error bars indicate mean ± SD. of indicated replicates.

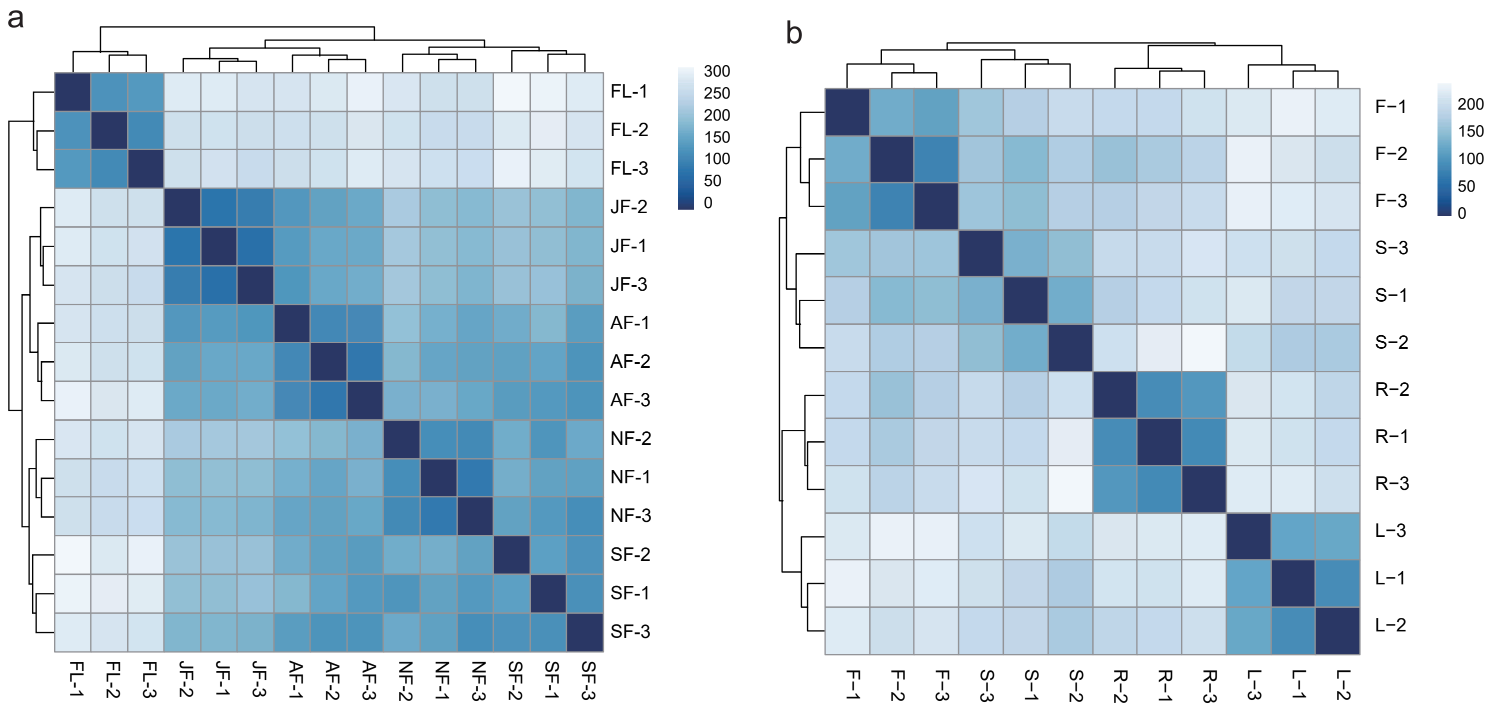

Supplementary Fig. 10 Sample to sample distance heatmap. a, heatmap of flower and different ripenning stages of fruits from July 'JF' to Noverber 'NF'. **b**, heatmap of different organs of sample collected in July.

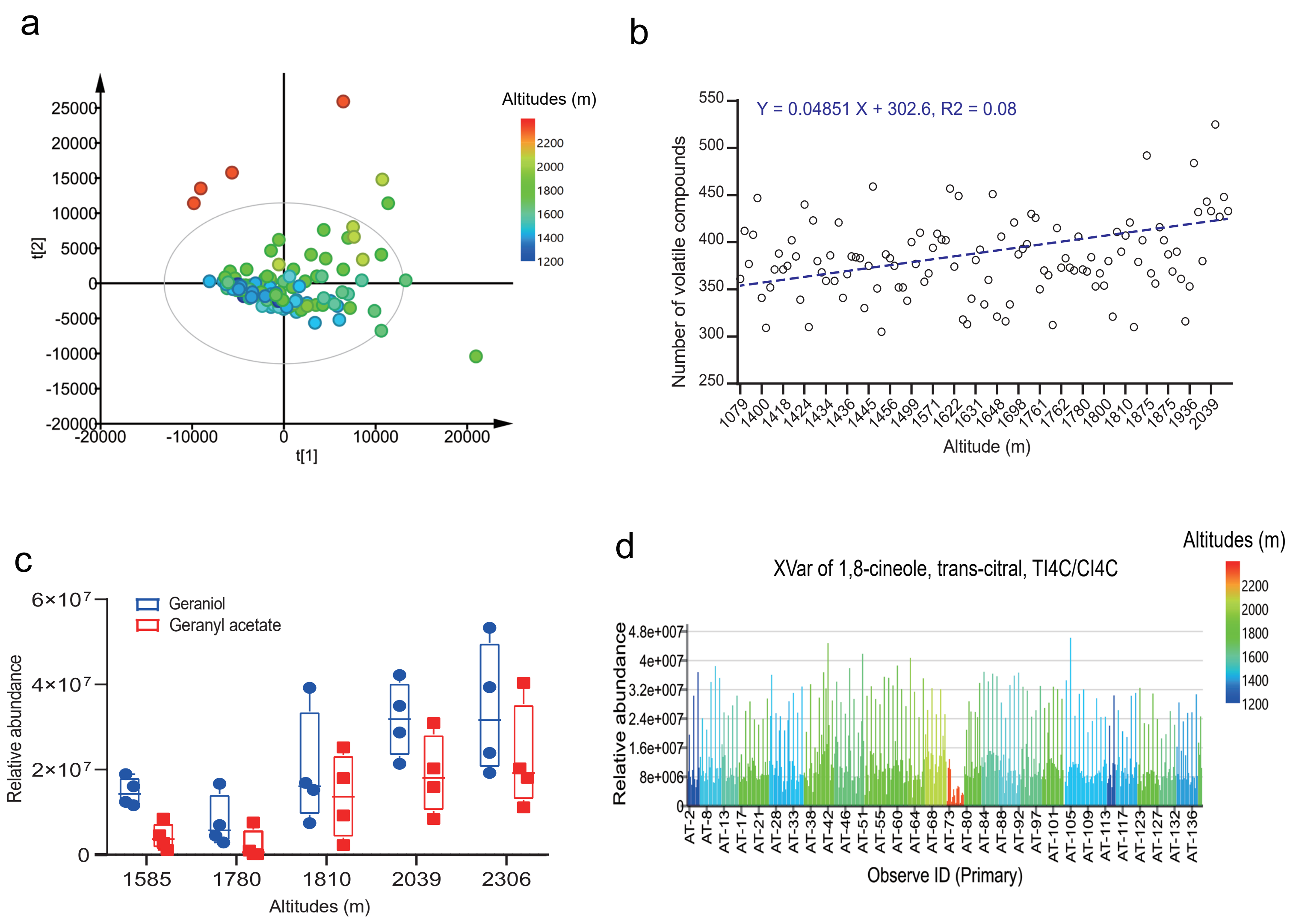

Supplementary Fig. 11 Trends in volatile aroma constituents of *A. tsao-ko* fruits from different altitudes measured with GC-MS. **a**, PCA scores plot of 119 fruits from different locations, colored by altitude. **b**, Relationship between number of volatile metabolites and altitude. The point marked with sample name was collected above 2000 m altitude. **c**, Variation in relative contents of geraniol and geranyl acetate by altitudes in three representative transects. **d**, Significant difference in compound levels from high altitude samples.

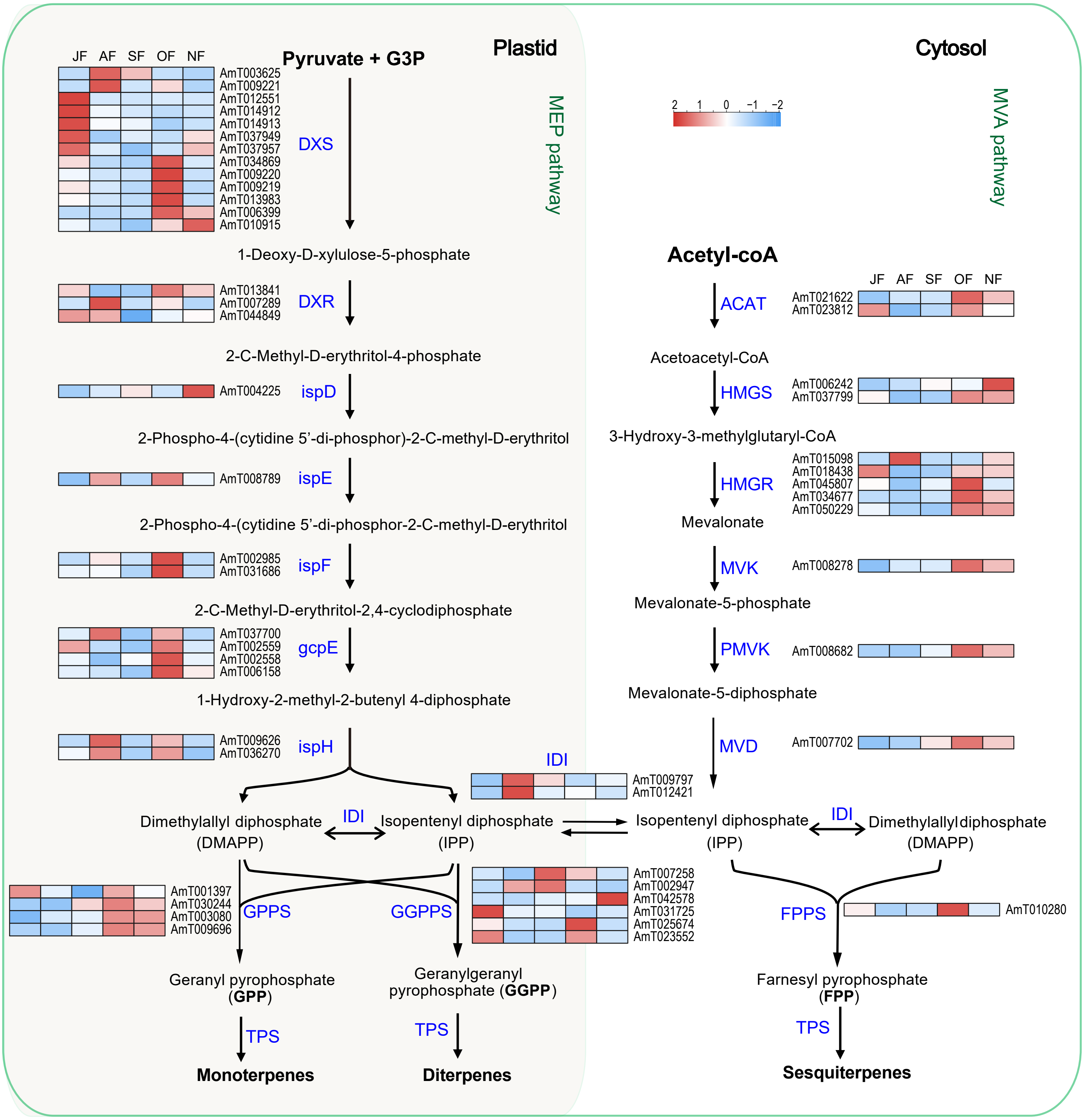

Supplymentary Fig. 12 Terpenoids biosynthesis in *A. tsao-ko*. The abbreviation of synthases (blue text) of terpenoids biosynthesis pathway refer to KEGG. The relative expression of related genes are displayed in heatmap.
