# Supplementary material for: Chromosome-level genome assembly of Amomum tsao-ko provides insights into the biosynthesis of flavor compounds
Source: Hortic Res. 2022 Sep 19;9:uhac211. doi: 10.1093/hr/uhac211 (PMC9719038; doi:10.1093/hr/uhac211)
Supplement: Web_Material_uhac211 [file web_material_uhac211.zip › Supplementary Information_R2.docx]

**Supplementary Information**

**Experimental part of main article**

**1. Genome analysis**

**Illumina library preparation and sequencing**

Genomic DNA was extracted from an *A. tsao-ko* sample. A total of 1.5 µg DNA per sample was used as input material for the DNA sample preparations. Sequencing libraries were generated using the Truseq Nano DNA HT sample preparation Kit (Illumina USA) following manufacturer’s recommendations, and index codes were added to attribute each sequence to its original sample. Briefly, the DNA sample was fragmented by sonication to about 350 bp in length, and then DNA fragments were end polished, A-tailed, and ligated with the full-length adapter for Illumina sequencing with further PCR amplification. Finally, PCR products were purified (AMPure XP system), and then libraries were analyzed for size distribution with an Agilent 2100 Bioanalyzer and quantified using real-time PCR. These libraries were constructed using the Illumina NovaSeq platform and 150 bp paired-end reads were generated with insert size around 350 bp.
**ONT library preparation and sequencing**

Genomic DNA was extracted by QIAGEN^®^ Genomic DNA extraction kit (Cat#13323, QIAGEN) according to the standard operating procedure provided by the manufacturer. Purity of the extracted DNA was determined with a NanoDrop^™^ One UV-Vis spectrophotometer (Thermo Fisher Scientific, USA); DNA was deemed sufficiently pure if OD260/280 was 1.8-2.0 and OD 260/230 was 2.0-2.2. Next, a Qubit^®^ 3.0 Fluorometer (Invitrogen, USA) was used to accurately quantify DNA. Then, long DNA fragments were selected using the BluePippin system (Sage Science, USA). Next, DNA was repaired, and DNA ends were prepared for adapter attachment. Then sequencing adapters supplied in the SQK-LSK109 kit were attached to the DNA ends. Finally, a Qubit^®^ 3.0 Fluorometer (Invitrogen, USA) was used to quantify the size of these library fragments. Finally, the flow cell of a Nanopore GridION X5/PromethION sequencer (Oxford Nanopore Technologies, UK) was primed and then loaded with the DNA library.

**Library preparation and chromosome assembly for Hi-C sequencing**

The Hi-C library was prepared using a modification of a procedure reported by Belton et al ^1^. In brief, freshly harvested leaves were cut into 2 cm pieces and vacuum infiltrated in nuclei isolation buffer supplemented with 2% formaldehyde. Crosslinking was stopped by adding glycine with additional vacuum infiltration. Fixed tissue was frozen in liquid nitrogen and ground to powder before re-suspending in nuclei isolation buffer to obtain a suspension of nuclei. The purified nuclei were digested with 100 units of Hind III and marked by incubating with biotin-14-dCTP. Biotin-14-dCTP from non-ligated DNA ends was removed by the exonuclease activity of T4 DNA polymerase. The ligated DNA was sheared into 300-600 bp fragments, blunt-end repaired and A-tailed, and then purified by biotin-streptavidin-mediated pull down. Finally, the Hi-C libraries were quantified and sequenced using the Illumina Hiseq platform (Illumina, San Diego, CA, USA).

Quality control of Hi-C raw data was performed using Hi-C-Pro (v2.8.0) as previously reported ^2^. First, low-quality sequences (quality scores<20), adapter sequences and sequences shorter than 30 bp were filtered out using fastp v0.12.6 (fastp, RRID:SCR_016962) ^3^, and then the clean paired-end reads were mapped to the draft assembly sequence using bowtie2 (v2.3.2) (bowtie2, RRID:SCR_005476) to obtain unique mapped paired-end reads ^4^. Finally, in combination with the validated Hi-C data, the LACHESIS (ligating adjacent chromatin enables scaffolding *in situ*) *de novo* assembly pipeline was used to produce chromosome-level scaffolds.

**Library preparation for transcriptome sequencing**

Three biological replicates were used for all *A. tsao-ko* samples. Total RNA was extracted and purified using TRIzol reagent (Invitrogen). Libraries with insert sizes ranging from 200 bp to 500 bp were produced and sequenced by Illumina NovaSeq 6000. We performed transcriptome sequencing for different tissues (from flower, stems, root and leaves) collected at the same growth stage, and for fruit collected in five different months from July to November (F7 to F11).

A total of 1 μg RNA per sample was used as input material for the RNA sample preparations. Sequencing libraries were generated using TruSeq RNA Library Preparation Kit (Illumina, USA) following manufacturer’s recommendations, and index codes were added to attribute each sequence to a specific sample. Briefly, mRNA was purified from total RNA using poly-T oligo-attached magnetic beads. First strand cDNA was synthesized using random hexamer primer and M-MuLV Reverse Transcriptase (RNase H-). Second strand cDNA synthesis was subsequently performed using DNA Polymerase I and RNase H. Remaining overhangs were converted into blunt ends via exonuclease/polymerase activities. After adenylation of 3’ ends of DNA fragments, Illumina adapters were ligated to prepare for hybridization. In order to preferentially select cDNA fragments 150–200 bp in length, the library fragments were purified with AMPure XP system (Beckman Coulter, Beverly, USA). Then PCR was performed with Phusion High-Fidelity DNA polymerase, Universal PCR primers and Index (X) Primer. Finally, PCR products were purified (AMPure XP system), and library quality was assessed on the Agilent Bioanalyzer 2100 system. The library preparations were sequenced on an Illumina Novaseq platform, and 150 bp paired-end reads were generated.

**2. The methods genome-wide association study (GWAS) analysis**

**Sampling for resequencing**

A total of 39 *Amomum tsao-ko* samples and 5 Zingiberaceae species (*Alpinia zerumbet*, *Amomum villosum*, *Amomum maximum*, *Amomum yingjiangense*, *Amomum koenigii*) were used for resequencing. All samples were collected from Yunnan provinces of China. After DNA extraction of each sample, a 350-bp insert library was constructed and paired-end sequencing was performed following the standard Illumina protocol. These libraries were sequenced by Illumina NovaSeq platform and 150bp paired-end reads were generated with insert size around 350bp.

**Read mapping and SNP calling**

The Illumina raw reads from re-sequenced samples were filtered using clean_adapter and clean_lowqual software (https://github.com/fanagislab/common_use), resulting in high-quality reads with an average error rate of < 0.01. Then, the high quality reads were aligned to the A. tsao-ko reference genome sequences by the Burrows-Wheeler Transform Alignment (BWA) software 53 package v0.7.5a with default parameters. Alignments for each sample were processed by removing duplicate reads using samtools software package v1.3 ^5^. The mpileup function in samtools was used to generate mpileup files for each sample. Bcftools-vcftools ^6^ was used to identify SNPs and small Indels. Several criteria were considered in SNP filtering: (1) a read mapping score higher than 40; (2) minimum coverage greater than 10.

**Population structure and genome-wide association analysis**

We used the biallelic loci SNPs presenting >95% individuals with a minor allele frequency of over 0.2 in the dataset were kept using software PLINK v1.9 ^7^ and used for population genetic structure analysis. The top two principal components were plotted against each other for the *A. tsao-ko* population. The associations between phenotypes (the metabolite contents of 39 samples from Supplementary Table 7) and SNPS was conducted using the mixed linear model ^8, 9^ by the software TASSEL version 3.0 ^10^. The top five principal components were used to build up the P matrix for population structure correction.

**3. Screening of candidate TPS genes and TFs related to terpene biosynthesis**

Candidate TPS genes were selected using genome annotation files. Then BLASTP, HMM and TERZYME searches, and all candidate TPS genes were further examined to confirm the presence of both PF01397 and PF03936 domains using the Pfam database and the NCBI’s conserved domains database (CDD). The candidate AmTTPSs were subjected to a phylogenetic analysis in raxmlGUI-2.0.0 using the maximum likelihood (ML) method with n = 1000 bootstrap replicates. The presentation of the resulting tree was augmented in iTOL (https://itol.embl.de/). The conserved motifs of AmTTPSs were discovered using MEME (<http://memesuite.org/tools/meme>). In addition, IBM SPSS 25.0 (Armonk, NY, United States) was used to obtain the Spearman correlation coefficients between terpene synthases and volatile terpenoids. Finally, using Cytoscape ^11^, a gene metabolite network map was constructed using results from the Spearman correlation (using only enzyme-compound pairs with correlation coefficient >0.6 and P-values <0.05). To uncover the TFs associated with terpene biosynthesis, we tested the differentially expressed AP2/ERF-, WRKY-, bZIP-, BHLH-, and MYB- family genes for their correlation with the candidate terpene synthase. This Spearman correlation analysis was done with expression data for TF genes and candidate monoterpene synthase (also in IBM SPSS 25.0). For pairs with Spearman correlation coefficients >0.8 and P-value <0.05, a putative TPSs-TFs network was constructed in Cytoscape. The sequences of those TFs positively correlated with monoterpene synthases were submitted online to the website ORF finder (https://www.ncbi.nlm.nih.gov/orffinder/) to determine the presence of full-length sequences. Then, the deduced amino sequences were submitted to the PfamScan website ([https://www.ebi.ac.uk/Tools/pfa/pfamscan/), to](https://www.ebi.ac.uk/Tools/pfa/pfamscan/),%20to) determine the presence of conserved domains.

**4. Analysis of metabolites constituents in *A. tsao-ko***

**Reagents for identification of secondary metabolites in *A. tsao-ko***

HPLC grade dichloromethane, acetonitrile, methanol and formic acid were purchased from J. T. Baker (Philipsburg, NJ, USA). The volatile standards 1,8-cineole, geraniol, (*E*)-2-octenal, α-pinene, nerol, geranyl acetate, geraniol, citral, trans-2 undecenal, (-)-α-terpineol, (*E*, *E*)-2,4-decadien-1-al, terpinen-4 ol, nonanal, (*E*)-2-decenal, nerolidol, vanillin, and linalool were purchased from Shanghai Macklin Biochemical Co., Ltd. China; cyclohexanone and C_8_-C_40_ *n*-alkanes were purchased from Sigma Chemical (St. Louis, MO, USA); 5-indanecarbaldehyde and 4-indanecarbaldehyde were obtained from Beijing Innochem Science & Technology co., LTD. China. The tsaokoin, tsaokoarylone, and hannokinol were purchased from BioBioPha Co., Ltd., Yunnan, P. R. China.

**Plant extraction for metabolites analysis**

Freeze-dried samples were ground to a powder under liquid nitrogen and passed through a 40 mesh screen. Each powdered sample (250 mg) was stirred with 5 mL dichloromethane containing cyclohexanone (internal standard) and sonicated for 30 min in a 20 °C water bath. The extracts were dried over anhydrous sodium sulfate and stored at -4 °C for 12 h. Then 0.2 mL of each extract was carefully aspirated, diluted 5 times to a concentration of 10 mg/mL (with 10 ppm cyclohexanone), and centrifuged at 8000 rpm for 30 s. Each supernatant was transferred into a separate auto-sampler vial for GCMS analysis.

Powdered sample (200 mg) was combined with 4 mL methanol (containing 10 ppm acetanilide and esculetin as internal standards) and sonicated at 20 °C for 30 min. A 1 mL aliquot of each extract was centrifuged at 10,000 rpm for 60 s prepare for the UPLC-QTOF-MS analysis. The filtrate was concentrated under vacuum by rotary evaporation to obtain a dry residue, redissolved with methanol to a concentration of 2 mg/mL of crude extract, and filtered (0.25 μm PTFE) to prepare for analysis.

**GC-MS analysis of volatile constituents.**

Volatile constituents were analyzed on a GC-MS instrument (Agilent 7890A and Agilent 5975B MSD, Agilent Technologies, USA) equipped with DB-5MS column (30 m × 0.25 mm × 0.25 µm, Agilent Technologies, USA) under the following conditions: helium as the carrier gas at 1.0 ml/min flow rate; injection volume 1 μl; injection temperature 230 °C. The oven temperature program was as follows: 60 °C held for 2 min, 60 °C to 280 °C at 6 °C/min; 280 °C held for 10 min. The MS was operated at an ionization voltage of 70 eV, an ion source temperature of 230 °C, and a mass range of 50–500 m/z. Compound identification was performed using a combination of relative retention index (RI), NIST mass spectral library matching and published mass spectra.

**UPLC-QTOF-MS analysis of non-volatile constituents.**

Metabolite analysis of samples was performed using an UPLC-QTOF-MS (Waters, Milford, MA, USA) as previously described ^12^. Chromatographic separation was performed on an ACQUITY UPLC^®^ BEH C18 column (2.1 mm × 50 mm i.d., 1.7 μm) and maintained at 40 °C. Mobile phases were: A, 0.1% formic acid in acetonitrile; B, 0.1% formic acid in water. The following linear elution gradient was used: 0 – 1.0 min, 10 – 15% A; 1.0–2.0 min, 15 – 30 % A; 2.0 – 4.5 min, 30 – 50 % A; 4.5 – 7.0 min, 50–80 % A; 7.0 – 9.0 min, 80–98 % A; 9.0 – 11.5 min, 98% A; 11.5 – 14.0 min, 10 % A. The flow rate was 0.3 mL/min. The auto-sampler was maintained at 12 °C, and the sample injection volume was 0.5 μL. Mass spectra were obtained on a Xevo G2 QTof-MS equipped with an electrospray ionization source and controlled by MassLynx 4.1 software. The sample capillary voltages were 3.0 and 1.50 KV in positive and negative modes, respectively, and the source temperature was maintained at 120 °C. Argon was used as the collision gas. N_2_ was used as desolvation gas (400 °C and 800 L/h flow rate) and cone gas (50 L/h). Data-independent acquisition mass spectrometry (MS^E^) was conducted to obtain the mass spectra within a *m/z* range of 100–1000 Da using a 0.5 s scan time. A low collision energy of 5 V was used to acquire precursor ion spectrum (MS), and a high energy ramp from 20 to 50 V was used to acquire fragmentation (MS^E^) data in the same analysis. An auxiliary pump delivered a reference solution of leucine encephalin (1 µg/mL in 50:50 ACN/water + 0.1% formic acid) for mass accuracy correction.

**Quantitative analysis of flavonoids and diarylheptanoids by UPLC-TQD-MS.**

Quantitative analysis of samples was performed on an ACQUITY™ UHPLC system couple with a triple-quadrupole Xevo TQD mass spectrometer. An ACQUITY UPLC® BEH C18 column (2.1 mm × 50 mm, 1.7 μm) was employed, and column temperature was maintained at 40 °C. A gradient elution with acetonitrile containing 0.1% formic acid (mobile phase A) and water containing 0.1% formic acid (mobile phase B) was performed as follows: 0–1.0 min, 10% A; 1.0–3.0 min, 10–50% A; 3.0–6.0 min, 50–95% A; 6.0–8.0 min, 95% A; 8.0–8.5 min, 95–10% A; 8.5–10.0 min, 10% A. Mobile phase flow rate was set at 0.3 mL/min. Sample injection volume was 2 μL. Mass spectrometric detection was performed on Xevo-TQD equipped with an electrospray ionization source. The capillary voltages were 2.5 and 1.5 KV in positive and negative modes, respectively, and the source was maintained at 150 °C. The collision gas was Ar, and N2 was used as the desolvation gas (400 °C and 700 L/h flow rate) and cone gas (50 L/h flow rate). All compounds were detected and quantified using multiple reaction monitoring (MRM) with a 0.025 s dwell time. TargetLynx software (Waters Corp.) was used for data analysis.

**5. Enzyme Assay and Product Analysis**

Crude enzyme assay was performed similar as previously describedwith modification ^13,14^. Briefly, fresh ripe fruits of *A. tsao-ko* were ground to a powder under liquid nitrogen. About 2 g of power was extracted with 8 mL buffer (0.1 mol/L Potassium dihydrogen phosphate/di hydrogen phosphate, PH 7.0). The extraction was placed for 1 h at 4°C, then filtered through filter paper under reduced pressure to obtain the *A. tsao-ko* protease extract. Next, 200 uL protease extract was placed to 2 mL tube, and added 1 mL of solution (1 ug/mL of compounds in 0.1 mol/L otassium dihydrogen phosphate/di hydrogen phosphate). The reaction was incubated at 37°C for 1 h and overlaid with 200 µL hexane. The hexane phase was extracted and used for GC-MS analysis as as described above.

1. **Chromosome Preparation**

The chromosome preparation was conducted as in a previously described method^15^. Briefly, the root tips of Amomom tsao-ko seeding with approximately 1 cm length were cut and pretreated with nitrous oxide gas for 2 h. The root tips were then fixed in ice-cold 90% acetic acid for 10 min and stored in 70% ethanol at -20°C. The root tips were washed in ice-cold 1 × citric buffer for 10 min. The root sections with actively dividing region were excised and incubated in an enzyme mixture containing 1% pectolyase Y 23 (Yakult Pharmaceutical, Tokyo, Japan) and 2% cellulose Onozula R10 (Yakult Pharmaceutical) for 2 h at 37°C. After digestion, the root sections were washed in ice-cold TE and 100% ethanol twice in sequence. The root sections were fine-broken with a needle and vortexed at 4000 rpm for 20 s. The cells were collected by centrifugation and resuspended in 100% acetic acid to prepare a cell suspension. The cell suspension was dropped onto glass slides in a moist box and dried. The slides were checked under a phase-contrast microscope.

**Reference**

1. Belton, J.M. *et al.* Hi-C: a comprehensive technique to capture the conformation of genomes. *Methods* **58**, 268-276 (2012).

2. Burton, J.N. *et al.* Chromosome-scale scaffolding of de novo genome assemblies based on chromatin interactions. *Nat Biotechnol* **31**, 1119-1125 (2013).

3. Chen, S., Zhou, Y., Chen, Y. & Gu, J. Fastp: an ultra-fast all-in-one FASTQ preprocessor. *Bioinformatics* **34**, i884-i890 (2018).

4. Langmead, B. & Salzberg, S.L. Fast gapped-read alignment with Bowtie 2. *Nat. Methods* **9**, 357-359 (2012).

5. Li, H. et al. The sequence alignment/map format and SAMtools. *Bioinformatics* 25, 2078-2079 (2009).

6. Li, H. A statistical framework for SNP calling, mutation discovery, association mapping and population genetical parameter estimation from sequencing data. *Bioinformatics* 27, 2987-2993 (2011).

7. Purcell, S. et al. PLINK: A tool set for whole-genome association and population-based linkage analyses. *Am J Hum Genet* 81, 559-575 (2007).

8. Yu, J. et al. A unified mixed-model method for association mapping that accounts for multiple levels of relatedness. *Nat Genet* 38, 203-208 (2006)

9. Zhang, Z. et al. Mixed linear model approach adapted for genome-wide association studies. *Nat Genet* 42, 355-360 (2010)

10. Bradbury, P. et al. TASSEL: software for association mapping of complex traits in diverse samples. *Bioinformatics* 23, 2633-2635 (2007)

11. Shannon & P. Cytoscape: a software environment for integrated models of biomolecular interaction networks. *Genome Res.* **13**, 2498-2504 (2003).

12. Li, P. *et al.* UPLC-QTOFMS(E)-guided dereplication of the endangered Chinese species *Garcinia paucinervis* to identify additional benzophenone derivatives. *J Nat Prod* **79**, 1619-1627 (2016).

13. Song Q. Study on ginger protease. Wuxi: Jiangnan University. (2009).

14. Wang, H. et al. An Integrative volatile terpenoid profiling and transcriptomics analysis for gene mining and functional characterization of AvBPPS and AvPS involved in the monoterpenoid biosynthesis in *Amomum villosum*. *Front Plant Sci* **9**, 846 (2018).

15. Li S. F, et al. The landscape of transposable elements and satellite DNAs in the genome of a dioecious plant spinach (*Spinacia oleracea* L.). *Mobile DNA* **10**, 3 (2019).
